# Why are enteric ganglia so small? Role of differential adhesion of enteric neurons and enteric neural crest cells.

**DOI:** 10.12688/f1000research.6370.1

**Published:** 2015-05-12

**Authors:** Benjamin N. Rollo, Dongcheng Zhang, Johanna E. Simkin, Trevelyan R. Menheniott, Donald F. Newgreen

**Affiliations:** 1 Murdoch Children's Research Institute, Royal Children's Hospital, Victoria, 3052, Australia

**Keywords:** Neural crest, intestine, enteric nervous system, cell adhesion, gangliogenesis, quail, chick

## Abstract

The avian enteric nervous system (ENS) consists of a vast number of unusually small ganglia compared to other peripheral ganglia. Each ENS ganglion at mid-gestation has a core of neurons and a shell of mesenchymal precursor/glia-like enteric neural crest (ENC) cells. To study ENS cell ganglionation we isolated midgut ENS cells by HNK-1 fluorescence-activated cell sorting (FACS) from E5 and E8 quail embryos, and from E9 chick embryos. We performed cell-cell aggregation assays which revealed a developmentally regulated functional increase in ENS cell adhesive function, requiring both Ca ^2+ ^-dependent and independent adhesion. This was consistent with N-cadherin and NCAM labelling. Neurons sorted to the core of aggregates, surrounded by outer ENC cells, showing that neurons had higher adhesion than ENC cells. The outer surface of aggregates became relatively non-adhesive, correlating with low levels of NCAM and N-cadherin on this surface of the outer non-neuronal ENC cells. Aggregation assays showed that ENS cells FACS selected for NCAM-high and enriched for enteric neurons formed larger and more coherent aggregates than unsorted ENS cells. In contrast, ENS cells of the NCAM-low FACS fraction formed small, disorganised aggregates.  This suggests a novel mechanism for control of ENS ganglion morphogenesis where i) differential adhesion of ENS neurons and ENC cells controls the core/shell ganglionic structure and ii) the ratio of neurons to ENC cells dictates the equilibrium ganglion size by generation of an outer non-adhesive surface.

## Introduction

The enteric nervous system (ENS) is derived from the neural crest (NC), a population of migratory mesenchyme cells originating in the dorsal neural tube. Most of the ENS arises from the caudal hindbrain (vagal) NC ( Yntema & Hammond, 1954), chiefly from the level of somites s3 to s5 ( Epstein *et al.*, 1994). These cells migrate to the nearby foregut, changing *en route* ( Simkin *et al.*, 2013) to become enteric NC (ENC) cells which are capable of exploiting the gut mesoderm. ENC cells migrate in the mesoderm along the midgut and hindgut to colonise the entire gastro-intestinal tract. This migration takes the form of intersecting narrow chains of motile ENC cells ( Druckenbrod & Epstein, 2005; Epstein *et al.*, 1991; Young *et al.*, 2004; Young *et al.*, 2014). Later the ENS comprises a network of numerous small, closely-spaced ganglia with many types of neurons and glia, with each ganglion connected via neurites and glial cells to other ganglia and to the smooth muscle and the mucosa ( Conner *et al.*, 2003; Epstein *et al.*, 1991; Fairman *et al.*, 1995). This distributed ENS network controls peristalsis as well as other gut activities ( Furness, 2012).

Developmental disorders of the structure, size and organization of the ENS ganglia have consequences for ENS function. Hypoganglionosis (fewer, smaller ganglia) is associated with persistent constipation, and mice heterozygous for the neurotrophic factor *GDNF* are hypoganglionic ( Flynn *et al.*, 2007). Defects involving an over-abundance of ENS cells and disturbance of their distribution are also known; hyperganglionosis in the form of enteric ganglioneuromas occurs in multiple endocrine neoplasia type 2 (MEN2B) syndrome as a result of constitutive activation of RET (the receptor for GDNF on ENS cells) and is accompanied by dysfunction of the ENS ( Takahashi *et al.*, 1999). Mouse *Zic2* mutants show an increased number of ENS neurons ( Zhang & Niswander, 2013). Other ENS disorganizations have also been described. In mice where *HAND2* is knocked out in ENS cells, the migration of ENC cells is not impaired but segregation into ganglia is abnormal ( D'Autreaux *et al.*, 2007; Lei & Howard, 2011). Mice with NC-specific knockout of β1-integrin show ENS ganglia which are morphologically different from normal ( Breau *et al.*, 2006).

 How ENC cell chain migration evolves into a ganglionated network, and how this is disturbed in some pathologies, is not well understood. NC derivatives elsewhere form relatively large ganglia, such as the dorsal root ganglia (DRG) and sympathetic ganglia. In the forming DRG and sympathetic ganglia, early differentiating neurons occupy the centre of cell aggregates with NC cells or glioblasts surrounding this core. This segregation is maintained in part by Notch signalling which suppresses neuronal differentiation in the peripheral cells ( Tsarovina *et al.*, 2008; Wakamatsu *et al.*, 2000). DRG and sympathetic ganglia are in stereotyped positions which are clearly related to and dependent on segmentally spaced cues from their mesodermal microenvironment ( Teillet *et al.*, 1987). Formation of each the NC-derived sympathetic ganglia, for example, relies on the scattered NC cells self-aggregating, driven innately by increased N-cadherin homophilic adhesion. This is combined with growth factor and cytokine attraction and repulsion from a patterned microenvironment to provide the positioning of each of the ganglia ( Kasemeier-Kulesa *et al.*, 2006; Kasemeier-Kulesa *et al.*, 2010). The size of the ganglia is also regulated. In the DRG, experimental NC overload and NC ablation suggest that the initial size of the ganglia reflects both the number of NC cells that give rise to them, together with early proliferation. The final size of the ganglia is adjusted in normal and NC cell overload conditions by population reduction by programmed cell death, due at least in part to logistic competition for survival factors. Conversely in NC cell under-supply conditions, compensatory mitosis occurs to increase the DRG cell population ( Barde, 1994; Kalcheim *et al.*, 1987; Zarzosa *et al.*, 2014).

The final ENS cell population is enormous but ENS ganglia are each very small and are located mainly in two narrow layers associated with the intestinal smooth muscle layers, the myenteric ganglia between the longitudinal and circular muscle layers and the sub-mucosal ganglia internal to the circular muscle. Typically migratory ENC cells occupy the myenteric layer early, prior to visible structural or molecular correlations including smooth muscle differentiation ( Newgreen & Hartley, 1995). A spatial association of the early ENS with the pre-existing intestinal vascular layer has been suggested but this is controversial ( Delalande *et al.*, 2014; Hackett-Jones *et al.*, 2011; Hatch & Mukouyama, 2015; Nagy *et al.*, 2009; Schrenk *et al.*, 2015; Young *et al.*, 2004). The sub-mucosal ENS cells originate later from this outer layer by centripetal migration (except in the avian hindgut), a process involving the response of DCC-expressing ENC cells to netrin produced by the endoderm ( Jiang *et al.*, 2003).

Cues governing the size, shape and location of each ENS ganglion are not well understood, but are clearly reliant on molecules from their local mesoderm microenvironment. With different-sized starting populations of ENC progenitors, resultant ENS ganglia achieve a similar, small size (compared to DRG) and a similar density of distribution ( Allan & Newgreen, 1980; Zhang *et al.*, 2010), suggesting some regulatory ability. Cell death is of great importance in most of the nervous system but is slight in the ENS ( Chalazonitis *et al.*, 2012). However, it does occur normally in the early ENS-fated population, at and before gut colonisation, well before the ganglia form. Inhibition of this early cell death leads later to an increase in the ENS cell population in those regions of the gut that are first colonised, and this increase takes the form of more densely distributed ganglia, but significantly the ganglia are of normal size and shape ( Wallace *et al.*, 2009).

The early ENS is strongly active mitotically ( Simpson *et al.*, 2007) in response to growth factors from the local environment. In particular the gut mesoderm cells produce GDNF (glial cell line-derived neurotrophic factor). GDNF is a survival factor and also initially a mitogen ( Hearn *et al.*, 1998) and chemotactic factor ( Young *et al.*, 2001) via its receptor RET on ENC cells. Later however, its role changes to an inducer of neuronal differentiation (which reduces proliferation) and axon growth. Down-regulation of GDNF during migration not only reduces ENC proliferation but also triggers premature neuronal differentiation ( Mwizerwa *et al.*, 2011). Endothelin-3 (ET-3) is also important at early stages, via its receptor EDNR-B on ENC cells. ET-3 is thought to dampen the differentiation response to RET activation, thereby prolonging the proliferative phase of ENC cells ( Hearn *et al.*, 1998; Wu *et al.*, 1999) and aiding distal intestinal colonisation ( Nagy & Goldstein, 2006). The mesodermal factor BMP2/4 is also important in ENS morphogenesis and differentiation. However, the ENS cell proliferation and neuronal differentiation response to BMP2/4 signalling by ENS lineage cells is complex, and may be influenced by factor concentration and by the time of exposure to alter proliferation and differentiation ( Chalazonitis *et al.*, 2004). Early BMP inhibition impairs aggregation of ENC cells into ganglia and leads to hypoganglionosis ( Goldstein *et al.*, 2005). BMP also has an effect on enteric gliogenesis, probably by priming ENC cells to respond to factors including Glial Growth Factor-2 ( Chalazonitis *et al.*, 2011). Furthermore, BMP increases the number of smooth muscle cells which are a major source of GDNF. In addition, BMP promotes polysialylation of NCAM on ENS cells which likely down-modulates cell-cell adhesivity ( Faure *et al.*, 2007); this may favour morphogenetic movement of ENS cells to allow structure changes such as establishment of the sub-mucosal plexuses.

The morphogenesis of the ENS is affected not only by mesoderm-derived soluble factors but also by structural elements such as extracellular matrix (ECM) ( Newgreen & Hartley, 1995). The shape, size and pattern of ganglia can be altered by manipulation of ECM adhesion properties. NC-restricted loss of β1-integrin receptor for ECM adhesion leads to larger, rounder, sparser and abnormally patterned ENS ganglia ( Breau *et al.*, 2006). It is assumed that a major β1-integrin ligand is the ECM adhesive molecule fibronectin. Interestingly, the morphogenetic disturbance of ENS gangliogenesis caused by genetic ablation of β1-integrin can be partially corrected by simultaneous deletion of the cell-cell adhesion molecule N-cadherin ( Broders-Bondon *et al.*, 2012).

ENS neurons, glia and ENC cells show differential labelling for various cell-cell adhesion molecules, and also differ from their surrounding mesodermal cells ( Hackett-Jones *et al.*, 2011; Nagy *et al.*, 2012). Here we describe cell-cell adhesion molecules in the ENS, and the roles of cell-cell adhesion in aggregation tests *in vitro*. In particular we wished to explore why the ENS ganglia are similar and small in size and why the initial cell disposition in ganglia has the pattern of central neurons surrounded by ENC progenitor cells.

## Materials and methods

### Embryo and tissue origin

Fertilised quail ( *Coturnix japonica*) and White Leghorn/Black Australorp cross chicken ( *Gallus gallus domesticus*) eggs were obtained respectively from Lago Game Supplies and Research Poultry Farm, Vic., Australia. Eggs were incubated at 38°C in a 60% humidity incubator. Embryos were staged according to the number of embryonic days (E) and Hamburger and Hamilton stages (HH) ( Hamburger & Hamilton, 1951). Animal ethics permission was obtained for the Royal Children’s Hospital Animal Ethics Committee, AEC677.

### Wholemount midgut preparation

The midgut (defined as the intestine caudal to the stomach to rostral to about half way along the caecum) was removed from quail (Q) embryos at half-day intervals from QE4.5 (HH25) to QE8 (HH34) and then at intervals to QE14 (HH43). These were fixed from times varying from 1 h to overnight in 4% PFA. Antigen retrieval of fixed specimens employed 10 mM citrate buffer pH 6 for 20 minutes at 95°C. Specimens were washed in phosphate-buffered saline (PBS) for 10 minutes, then blocked and permeabilised overnight with 1% horse serum (CSL, Melb., Aust.) and Triton X-100 (Sigma-Aldrich, USA) at 0.1% in PBS. These were then incubated for 1–2 days at 4°C sequentially in primary and secondary antibodies (see Supplemental Table T1) prepared in blocking/permeabilising solution. Between treatments, the specimens were washed extensively in PBS. Gut tissue and aggregate specimens were mounted in Vectashield antifade reagent (Vector Laboratories, Inc., CA, USA) between two coverslips with coverslip spacers.

### Counts of myenteric ENS cells in wholemounts

Four to eight areas of 100×100 μm were selected along the midguts from QE4.5 to QE8.5 and cell counts of Hu ^+ve^ and SoxE ^+ve^ cells were made from optical sections of the myenteric plexus. Cells outside this layer, such as submucosal ENS cells, were not counted.

### Cell dissociation

**Supplemental Figure S1.**
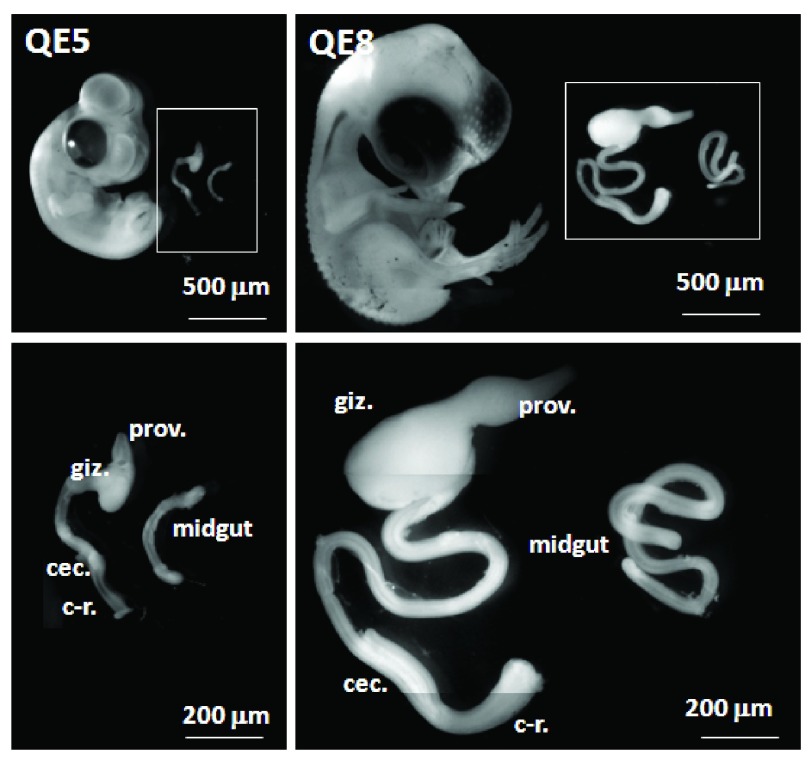
QE5 and QE8 midgut ENS donor tissue. Intestine (midgut) caudal to the gizzard (giz.) to about half way along the cacum (cec.) was obtained. This was removed from E5 (about HH27) and E8 quail embryos (about HH34-35). HH34-35 midgut was also obtained from 9-day chick embryos. The intestine was cut at the dorsal border of the mesentery to exclude cells of the Nerve of Remak. The tissue used did not include the cecal root because here the Nerve of Remak is applied to the gut so closely that contamination is unavoidable. C-r = colo-rectum, prov. = proventriculus

The midgut was removed from QE5 (HH27) and from QE8 embryos and 9-day chick embryos (ChE9), about HH34-35 ( Supplemental Figure S1). The intestinal tissue pooled from 15–60 embryos was digested for 35 minutes at 37 ^o^C in Ham’s F12 media (Gibco Cell Culture, Invitrogen, USA) with 0.5% w/v Dispase II (Roche, USA) and 0.05% w/v CLSAFA Collaganase (Worthington, USA). To disrupt cadherin-based cell interactions ethylenediaminetetraacetic acid (EDTA; Sigma-Aldrich, USA)) was added to a concentration of 1 mM for a further 10 minutes. The tissue was mechanically triturated and the cell suspension was washed in F12 media with 5% BSA. Cells from mid-trunk dorsal root ganglia (DRG) from the QE8 embryos were dissociated in the same way.

### Fluorescent labelling and fluorescence-activated cell sorting (FACS)

**Supplemental Figure S2.**
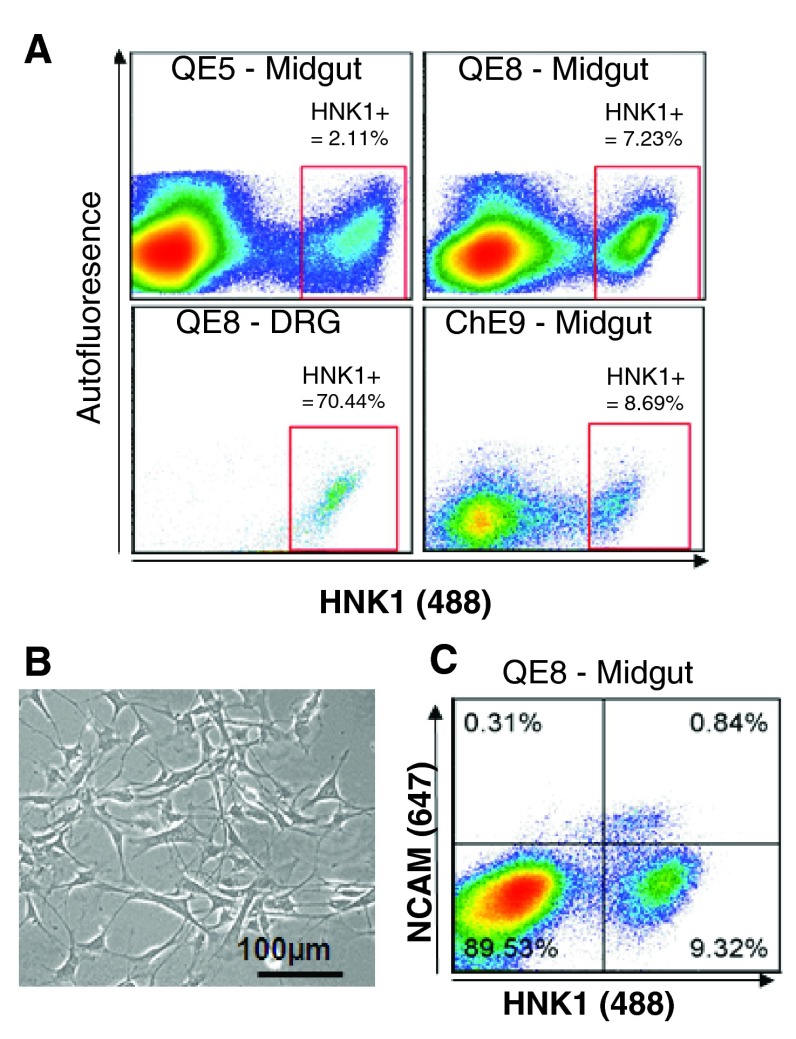
FACS of neural crest cells from avian tissue. Following tissue digestion, single cells were fluorescently labelled with HNK1 and HNK1/NCAM and cells sorted by FACS. ( **A**) Scatter plots show typical results of sorting embryonic tissue from quail embryonic day 5 (QE5), QE8, and chicken embryonic day 9 (ChE9). The typical percentage yields of HNK1 ^+ve^ cells are shown following FACS of single cells from the midgut and dorsal root ganglia (DRG). ( **B**) QE5 MG HNK1 ^+ve^ cells plated on laminin show the characteristic multipolar morphology of ENC cells. ( **C**) QE8 midguts were digested and single cells fluorescently labelled with HNK1 (Alexa 488) and NCAM (Alexa 647). The scatter plot shows that the majority of NCAM ^+ve^ cells also express HNK1. NCAM ^+ve^/HNK1 ^+ve^ are able to be differentially sorted from the NCAM ^-ve^/HNK1 ^+ve^ population.

Intestinal and DRG cells were labelled in suspension with mouse anti-HNK-1 IgM antibody (1/50 volume of supernatant; hybridoma maintained at MCRI) followed by secondary labelling with goat anti-mouse IgMμ Alexafluor 488 antibody ( Supplemental Table 1). In some cases mouse anti-NCAM was also included followed by goat anti-mouse IgG Alexafluor 647 (see Supplemental Figure S2). Cells were filtered through a 30 μm strainer (BD-Falcon, USA) and propidium iodide (Sigma-Aldrich, USA) was added (final concentration 10 μg/ml) to detect dead cells. Cells positive for Alexa 488 fluorescence which also excluded propidium iodide were sorted using a MoFlo cell sorter (MoFlo, USA). About 2–3% of the dissociated QE5 and 5–10% of QE8/ChE9 midgut cells were selected by this process ( Supplemental Figure S2). Each QE5 midgut segment provided 1900–2900 HNK-1+ve cells (range from 4 runs, total 268 midgut segments). Each QE8 gut segment yielded about 40–50,000 HNK-1+ cells (range from 4 runs, total 128 midgut segments), and the ChE9 about 50–60,000 HNK-1+ cells (range from 2 runs, total 33 midgut segments). HNK-1 FACS of QE8 DRG as expected produced a yield of >70% of the dissociated cells being HNK-1 ^+ve^.

### Cell identification and FACS validation

After selection by FACS, HNK-1 ^+ve^ and HNK-1 ^-ve^ cells (as well as unselected cells) were examined by q-PCR for the NC marker Sox-10 and the neuronal marker Hu-D, and by cell culture.

Total RNA was isolated from cells using the RNeasy mini kit (Qiagen, USA) and contaminant genomic DNA removed with *DNA-free* reagents (Ambion, USA). Primer sequences were designed using Primer3 ( http://frodo.wi.mit.edu/primer3/) and are listed below.

**Primer sequences**

GAPDH: 5’-TTATCATCTCAGCTCCCTCAGC-3’, 3’-AAGTTGTCATGGATGACCTTGG-5’;

SOX10: 5’-AGGAAATTGGCTGACCAGTACC-3’, 3’-GTCCTTCTTGTGCTGCATCC-5’;

HU-D: 5’-ACAGATGACAGCAAAACCAACC, 3’-ATTTTGTCTCTCACGAGCTTGC-5’.

**Supplemental Figure S3.**
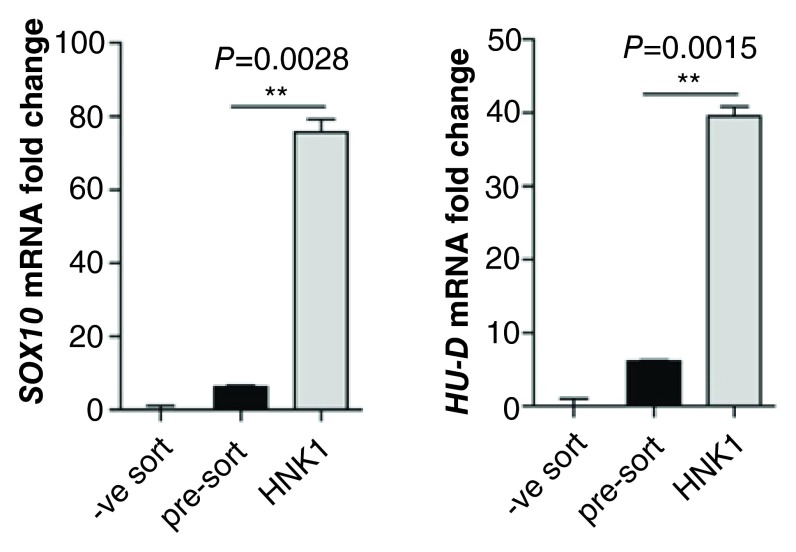
Qualitative (q) PCR of HNK1 sorted cells. To assess the enrichment for ENC markers following FACS for HNK1, QE8 midgut-derived cells were assessed for expression of SOX10 (a NC marker) and Hu-D (a neuronal marker). Cell populations analysed by qPCR were cells which were not sorted (pre-sort), sorted cells which were not HNK1 ^+ve^ (-ve sort) and HNK1 ^+ve^ sorted cells (HNK1). HNK1 ^+ve^ cells showed a significant increase in expression of the markers tested when compared with pre-sorted cells indicating successful enrichment of ENC cells by FACS.

For quantitative reverse transcription and polymerase chain reaction qRT-PCR, oligo-dT primed cDNA was synthesised from 200 ng total RNA using Murine Moloney Leukaemia Virus reverse transcriptase (Promega, USA). qRT-PCR was performed on an ABI Prism ^®^ 7500 Real Time PCR System using SYBR green master mix (Applied Biosystems, USA) according to the manufacturer’s protocols. Relative gene expression values were obtained by normalization to the reference gene GAPDH using the −2 ^ΔΔCt^ method, where −2ΔΔCt = ΔCt sample−ΔCt calibrator as described ( Peterson *et al.*, 2010). All fold changes were calibrated to the negative sort population. Results are shown in Supplemental Figure S3.

**Supplemental Figure S4.**
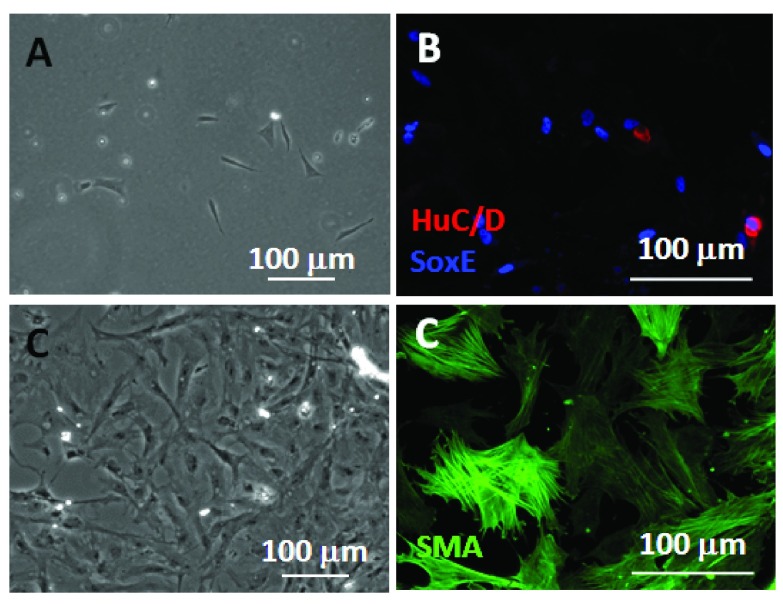
FACS for HNK-1 selected NC-derived cells from gut mesoderm cells. **A**. HNK-1 ^+ve^ cells plated for 18 h on laminin (phase contrast). **B**. HNK-1 ^+ve^ cells at 66 h *in vitro* showed NC markers: HuC/D (neurons) and SoxE (ENC cells). **C**. HNK-1 ^–ve^ cells plated for 66 h had flat fibroblastic morphology (phase contrast). **D**. Nearly all HNK-1 ^–ve^ cells plated for 66 h were highly flattened and exhibited smooth muscle actin (SMA) labelling.

For cell culture substrates, HLA Terasaki-plates (10 μl wells; Greiner Bio-One, Sigma M6062) were coated with human plasma fibronectin (FN; 20 μg/ml in PBS, 2 h; Roche 11051407001) or rat laminin-1 (LN; 50 μg/ml in PBS, 2 h; Roche 1124321700). HNK-1 and NCAM FACS-selected quail E8 midgut ENS cells were plated at 3000 cells/well into the above wells in Ham’s F12 with 1–10% heat inactivated fetal calf serum (FCS; Thermo-Fisher, USA) plus 0.5% BSA, and penicillin/streptomycin (pen/strep; Sigma-Aldrich, USA). In addition, the HNK-1 ^-ve^ cells were also plated in the same way. Cells were fixed and immunolabelled at 18 h to 66h *in vitro*, as for gut wholemounts, except that Terasaki cultures were not antigen retrieved (see Supplemental Figure S4).

Both q-PCR and culturing of FACS sorted cells indicated that the HNK-1-based sorting accurately selected for virtually all cells in the gut that expressed NC and neuronal markers.

### Short term cell aggregation assays

After cell dissociation and FACS analysis, the HNK-1 ^+ve^ cells remained for 1 h at 37°C in cell culture medium of F12 with 2% heat inactivated FCS, 0.5% BSA and pen/strep, to recover cell-cell adhesive potential ( Steinberg *et al.*, 1973; Takeichi, 1977). A low degree of aggregation occurred in the recovery period, indicated by a 6–15% reduction in particle number from the count recorded at the time of FACS. In three dissociation runs Calcein AM (1/4000; Invitrogen/Molecular Probes) was added for 20 min to reveal live cells, with the cells then centrifuged into fresh medium. This indicated that about 90% of cells were alive at this stage. The cell suspension was then aliquoted into Eppendorf tubes (100 μl cell suspension/tube) with particle density adjusted to 0.3–0.5×10 ^6^ cellular particles/ml. A minimum of three replicate tubes were prepared for each assay, and each assay was repeated at least twice. In parallel tubes ethylene glycol tetraacetic acid (EGTA) was added to 1 mM; this chelates Ca ^2+^ and therefore prevents cadherin-dependent cell-cell adhesions. The tubes were then incubated on a rotating platform (120 rpm, radius 1 cm) at 37°C. At t=0, 15, 30, 60 and 120 min., 10 μl samples were withdrawn from each tube and the particles were counted (see below). A particle was defined as a single cell or group of contacting cells of any size. Cell aggregation was indicated by a decreasing particle number.

### Longer term cell aggregation assays

Cells were allowed to aggregate as above, but with aggregation time of 2h, 4h, 6h, 18h and 48h. Rotation rates of 150, 120, 100, 75 and 0 rpm were tested. Starting cell densities were varied from 0.167×10 ^6^ to 1.0×10 ^6^ particles/ml. Aggregation assays were also performed with the inclusion of BrdU (Amersham-GE Healthcare, USA) for 4 h prior to fixation. At the end of the incubation period, aggregates were collected for imaging and image measurement by allowing them to settle in the Eppendorf tube for 5 min then removing the bottom 20 μl of medium plus aggregates. This was placed as a standing drop on a non-TC Petri dish which was oscillated at 80 rpm for 5 minutes to centralise the aggregates, which were then imaged. For fixation, 200 μl of 4% PFA in PBS was added to each Eppendorf tube. After overnight fixation, the fixative was washed out with PBS prior to immunolabelling as for midgut wholemounts.

### Imaging, cell and aggregate counting and evaluation

Samples were screened using an Olympus IX70 microscope (Olympus Optical Co., Tokyo, Japan), under selective Texas Red, FITC and AMCA filters, and by phase contrast. Images were recorded using a Spot Monochrome camera model 2.1.1 with Image-Pro Plus 4.5 (MediaCybernetics, Silver Spring, MD, USA). Confocal imaging was prepared on a Leica TCS SP2 with image processing via Leicalite and Image-Pro–Analyser 6.1 (MediaCybernetics). For short-term aggregation assays, particles (cells and cell groups) at each time point were counted in a haemocytometer chamber with 10–20 microscope field images each of 1.14 mm ^2^ recorded using an Olympus IX70 microscope (Olympus Optical Co., Tokyo, Japan) with 10× objective. Particle counts were made from these images by operators blinded to the assay conditions. For the long term aggregation assays, aggregate diameters were measured from phase contrast images (×20 objective) of at least 50 aggregates per treatment and time. Aggregates were chosen for measurement on the basis of roundness and defined edges, and very small and or loose cell clusters and single cells were ignored.

### Statistical analysis

Unless specified, data were expressed as mean± standard error of mean (SEM). All statistical tests were performed using GraphPad Prism version 6. A difference between two groups was determined using a two-tailed Student’s t-test and for nonparametric data Mann-Whitney test was used. For differences among multiple groups, statistical comparisons were performed using one-way analyses of variance (one-way ANOVA) followed with Fisher’s LSD post-test. A p-value of <0.05 was considered significant.

## Results and discussion

### The developing ENS *in vivo* has a constant ENC cell/neuron ratio

**Figure 1.**
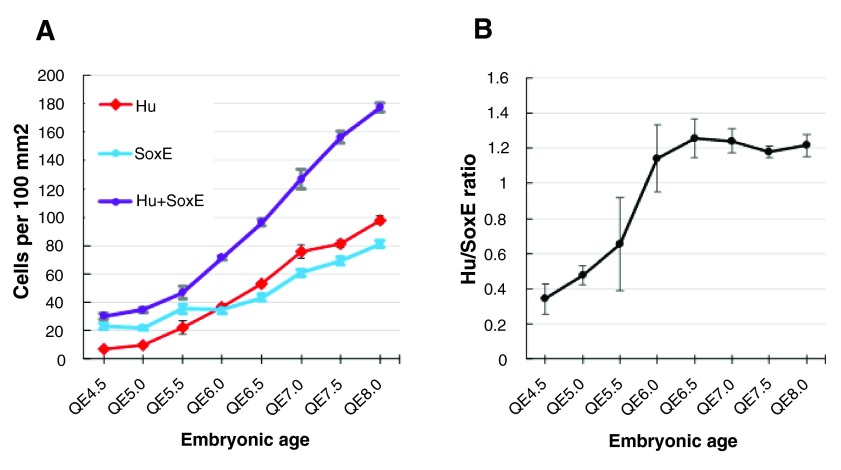
The density of neurons and ENC cells increases in the quail embryo midgut myenteric plexus, but the ratio stabilises. **A**. The density (number per 100×100 μm area) of neurons (Hu; red), ENC cells (SoxE; blue) and total ENS cells (Hu plus SoxE; purple) increases from QE4.5 to QE8. **B**. The ratio of neurons to ENC cells stabilises by E6. Error bar=SEM.

The sparse ENS cell population in the nascent myenteric plexus of the midgut was dominated by SoxE ^+ve^ ENC cells. The total ENS cell density (cells/unit plexus area) continued to increase over the period QE4.5 to QE8 ( Figure 1) and the plexus area increased by exponential gut growth ( Binder *et al.*, 2008). From about QE6, correlating with the assembly of ENS cells into coherent groups, the proportion of Hu ^+ve^ neurons increased to reach a ratio of 1.2:1 Hu ^+ve^: Sox ^E+ve^ cells. This ratio was maintained until at least QE8 ( Figure 1). The constancy of the ratio of Sox ^+ve^ cells to Hu ^+ve^ cells while the population number and density increased suggests an effective co-ordinate control between neurons and ENC cells.

Inhibition of Notch activity in mice by NC cell-specific knockout of the *Pofut1* gene, and Notch inhibition by DAPT in mouse and human enteric neurospheres *in vitro* ( Okamura & Saga, 2008; Theocharatos *et al.*, 2013) led to loss of Sox10 ^+ve^ ENC cells and a bias towards enteric neuron differentiation. This strongly suggests that after cell aggregation has been achieved by morphogenetic cell re-arrangements, the Notch system forms part of the intercellular signaling agency maintaining the ENC cell/neuron balance in the developing ENS.

Raw data for Figure 1 MG1 neural cell count ( Rollo *et al.*, 2015a).

### ENS ganglia undergo progressive morphogenesis *in vivo*

**Figure 2.**
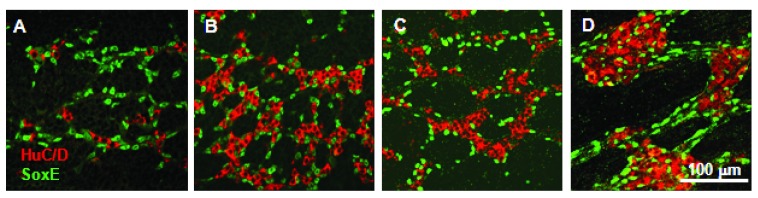
Gradual aggregation of ENS cells form ganglia in the embryonic quail midgut. **A**. QE5 midgut with chains of SoxE ^+ve^ ENC cells and a smaller number of scattered Hu ^+ve^ neurons. **B**. QE6 midgut with relatively more neurons in small groups, with adjacent ENC cells. **C**. QE8 midgut with coherent ENS neuron groups surrounded by SoxE ^+ve^ cells. **D**. QE14 midgut with large ENS ganglia with SoxE ^+ve^ cells both around the ganglia and in the ganglia mixed with the neurons, and also distributed along interganglionic tracts. Images are single confocal optical sections through the myenteric plexus.

In the quail embryonic midgut at HH27 (QE4.5 to 5), shortly after arrival of vagal ENC cells at HH25/6 (QE4.25), the relatively sparse ENS cell population was mainly SoxE ^+ve^ ENC cells (Hu-ve) distributed in chains ( Figure 2A). The number of Hu ^+ve^ neurons (SoxE ^-ve^) increased in the nascent myenteric plexus and they commenced forming clusters by QE6 ( Figure 2B). Later (QE8), the Hu ^+ve^ cells and the SoxE ^+ve^ cells formed co-aggregates with almost all the SoxE ^+ve^ cells segregated to the periphery of each neuronal cluster ( Figure 2C). The ENS cell aggregates increased in size and developed increasingly smooth borders and by QE14 the SoxE ^+ve^ cells were found not only surrounding the neuron groups but also between the individual neurons, as well as along axon tracts ( Figure 2D). In other NC-derived ganglia like the DRG a similar sequence of events, but chronologically earlier, has been observed, with the late-appearing intraganglionic cells being differentiated ganglionic glia ( Henion *et al.*, 2000).

We sought an explanation in differential cell-cell adhesion ( Steinberg, 2007) for the progressive aggregation of ENS cells into ganglia, with internal neurons and a shell of ENC cells. Time-lapse microscopy in mouse intestine has revealed that ENS cells (both neurons and ENC cells) are motile for a considerable period after the initial colonization phase ( Hao *et al.*, 2009; Young *et al.*, 2014). It can be imagined that such motile ENS cells might, by differential adhesion, finally collect together to form few very large ganglia; we therefore also sought reasons for the ENS forming only small ganglia.

### HNK-1 FACS effectively selects for NC-derived cells

Unsorted dissociated midgut cells showed modest NC (Sox10) and neuronal markers (Hu) by q-PCR. The HNK-1 ^+ve^ sorted moiety showed high levels of these neural sequences whereas the HNK-1 ^-ve^ moiety had very low expression ( Supplemental Figure S3). HNK-1 ^+ve^ sorted cells plated on fibronectin or laminin surfaces showed neural immunoreactivity, including SoxE (recognizes Sox9 and Sox10), HNK-1, HuC/D, Tuj1 and E-C8. A few cells (<2.5% at 18 h *in vitro*) were negative for neural markers but fibroblast-like in appearance and smooth muscle actin (SMA) ^+ve^. We regard these as contaminating gut mesoderm cells. In contrast HNK-1 ^-ve^ cells when plated were virtually entirely fibroblast-like in appearance and SMA ^+ve^ ( Supplemental Figure S4). This cell sorting procedure therefore provides highly enriched ENS cells for performance of cell aggregation assays.

### ENS cell clusters *in vitro* are due to cell aggregation, not proliferation

Dissociated HNK-1 ^+ve^ midgut ENS cells in low serum aggregation assays rapidly formed clusters which were relatively small and uniform. We examined ENS cell aggregates (N=7) at 22 h with confocal microscopy after 4 h bromodeoxyuridine (BrdU) exposure, and examined 18 h aggregates (N=6) with phosphohistone-H3 antibody and detected no cells labeled by these markers of proliferation. We conclude that there was little or no cell proliferation *in vitro*, and therefore the cell clusters under these conditions are due to cell aggregation.

### ENS cell aggregation indicates several adhesive mechanisms are operative and increase with age

Avian ENS cells in previous studies showed immunoreactivity for N-cadherin and NCAM and also for Ng-CAM (L1CAM) at the stages equivalent to the early stage shown above ( Hackett-Jones *et al.*, 2011; Nagy *et al.*, 2012). Ng-CAM soon became almost undetectable while N-cadherin and NCAM labelling became more intense on both SoxE ^+ve^ and Hu ^+ve^ cells. By QE8 in the midgut NCAM was clearly more strongly labelled on the Hu ^+ve^ neurons compared to the SoxE ^+ve^ cells ( Hackett-Jones *et al.*, 2011). This suggests that there may be a general increase in cell-cell adhesion in the ENS and a further increase in adhesion between neurons.

**Figure 3.**
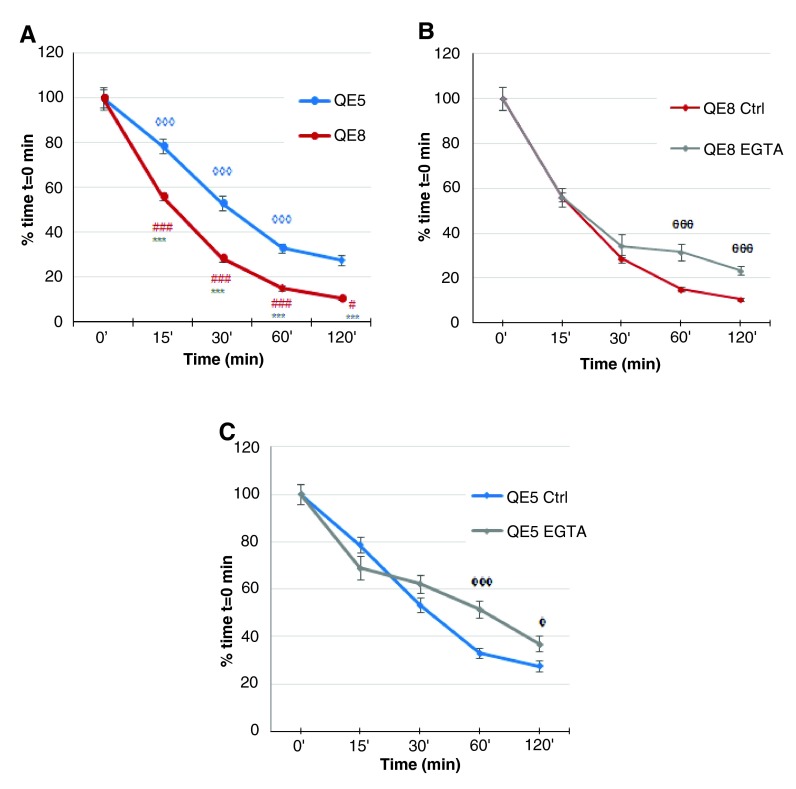
Initial aggregation kinetics of HNK-1 ^+ve^ midgut ENS cells are stage and cadherin dependent. Aggregation was indicated by decrease in particle count and is expressed as % of time t=0 min. **A**. QE5 and QE8 ENS cells aggregated continuously, with particle count at each time point significantly less than at the previous time point (0 min vs 15 min, 15 min vs 30 min, 30 min vs 60 min and 60 min vs 120 min) (◊◊◊ p<0.001 for QE5; # p=0.0455, ### p<0.001 for QE8), except for QE5 at 60 min vs 120 min, where the particle counts were not significantly different. In addition, aggregation was greater for QE8 ENS cells compared to QE5 ENS cells (*** p<0.001 QE8 vs QE5 at each time point), consistent with a developmentally increasing adhesive capacity. **B**. With EGTA, early aggregation (0 min, 15 min and 30 min) of QE8 ENS cells proceeded rapidly and was not significantly different from particle counts in control medium at the same time points. At later time points (60 min and 120 min) particle counts with EGTA were significantly greater than from the same time points in control medium (θθθ p<0.001 EGTA vs Ctrl). This strongly indicates that early aggregation events in these assays are largely calcium-independent but after about 30 min aggregation is dependent on cadherin function. **C**. QE5 ENS cells showed a similar early rate of decline in particle number with EGTA medium at matched time points of 0 min, 15 min and 30 min. Later, particle number decline decreased in EGTA (φφφ, p<0.001 EGTA vs Ctrl at 60 min; φ p=0.0225, EGTA vs Ctrl at 120 min;). This indicates that for QE5 ENS cells early aggregation is largely calcium-independent but further aggregation requires cadherin function. Error bar=SEM.

**Figure 4.**
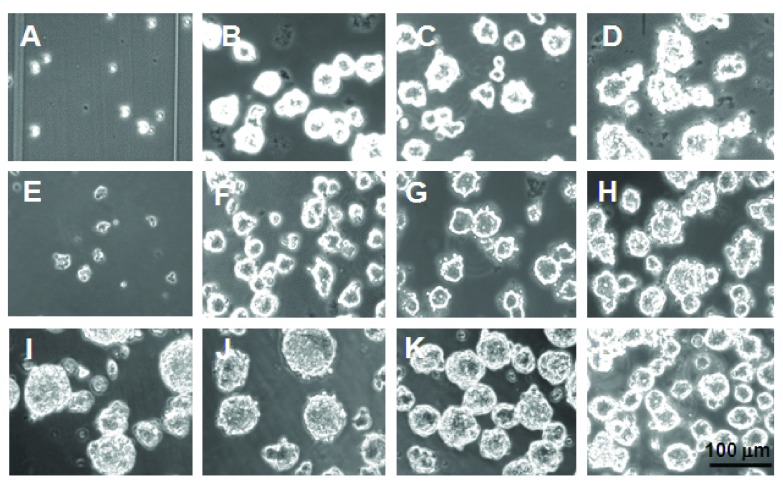
HNK-1 ^+ve^ ENS cells in rotating culture form aggregates. ENS cells at 0 h ( **A**), 4 h ( **B**), 18 ( **C**) and 48 h ( **D**) show rapid aggregation without the later formation of super-aggregates. The importance of cadherins is shown by Ca ^2+^-chelation with 1 mM EGTA, which reduced aggregate formation at 18 h ( **E**). **F**– **H**. Altering the rotation speed (0 rpm ( **F**), 75 rpm ( **G**) and 150 rpm ( **H**)) had only slight effect on aggregation at 18 h. **I**– **L**. Increasing the initial ENS cell density (0.167×10 ^6^ cells/ml ( **I**), 0.33×10 ^6^ cells/ml ( **J**), 0.67×10 ^6^ cells/ml ( **K**), 1.0×10 ^6^ cells/ml ( **L**)) resulted in a decrease in the aggregate size by 18 h.

The short-term rotating cell aggregation assays indicated that dissociated QE5 and QE8 midgut ENS cells adhered progressively ( Figure 3A, Figure 4A–D), but aggregation was faster and more complete in cells from the older embryos. This confirms a developmental increase in ENS cell cohesion. QE5 and QE8 ENS cell aggregation occurred over the first 30 minutes at the same rate with Ca ^2+^ chelation (i.e. 1 mM EGTA) as with normal medium but later the level of cell aggregation was impaired ( Figure 3B, C, Figure 4E). The sensitivity to EGTA showed involvement of Ca ^2+^-dependent (i.e. cadherin) mechanisms, but the residual aggregation indicated Ca ^2+^-independent mechanisms (such as NCAM) were operative as well, and suggests that the initial phase of adhesion in these conditions was largely due to Ca ^2+^-independent adhesion. This is in accord with the immunoreactivity for both N-cadherin and NCAM *in situ* noted above and in these cells *in vitro* ( Figure 6A, B).

Raw data for Figure 3A, B and C Aggregates at QE5 and QE8, control and with EGTA. Aggregation was indicated by decrease in particle count and is expressed as % of time t=0 min ( Rollo *et al.*, 2015b).

### ENS cell aggregation is progressive

**Figure 5.**
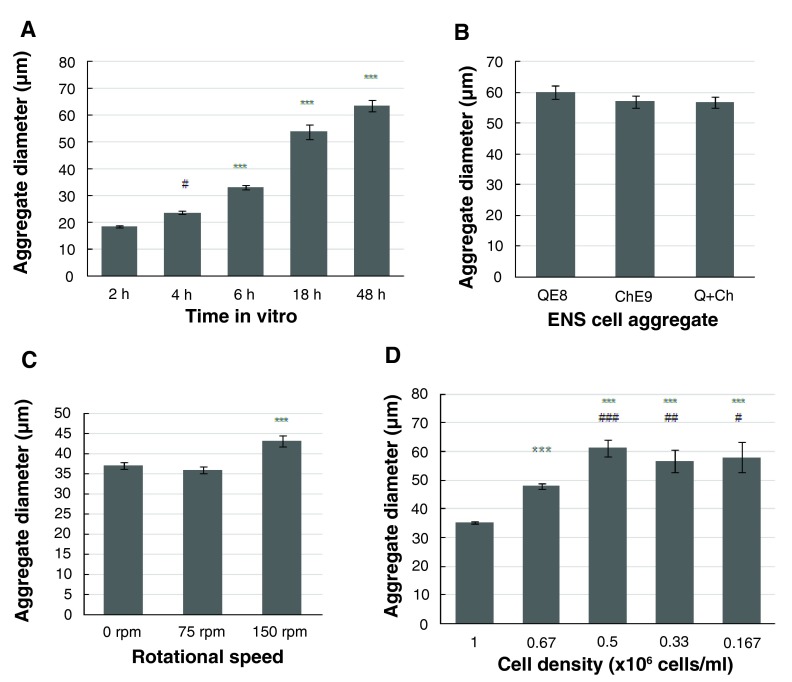
Histograms of the size of ENS cell aggregates in rotation cell-cell adhesion assays. **A**. QE8 cell aggregates gradually increased in diameter from 0–48 h *in vitro*. (# p=0.0229, 4h vs 2h; ***p<0.001 for all other times). Starting cell density 0.3×10 ^6^ cells/ml. **B**. ENS cells from QE8, ChE9 and mixed populations (Q+Ch) showed identical aggregation at 18 h *in vitro*, with no significant difference (Q:Ch p=0.29; Q:Q+Ch p=0.23; Ch:Q+Ch p=0.93;). Starting cell density 0.3 ×10 ^6^ cells/ml. **C**. Aggregate diameter attained at 18 h *in vitro* was slightly larger at the highest rotational speed (*** p<0.001, 150 rpm vs 0 and 75 rpm). Starting cell density 0.5×10 ^6^ cells/ml. **D**. Aggregate diameter at 18 h *in vitro* was starting cell density-related. Starting cell density at 1×10 ^6^ cells/ml produced aggregates of least diameter. With the gradually reduced starting cell density, the aggregate diameter increased (relative to 1×10 ^6^ cells/ml: *** p<0.001; relative to 0.67×10 ^6^ cells/ml: # p=0.0186, ### p<0.001). However, this increase plateaued, with no significant difference in aggregate diameter at 0.167, 0.33 and 0.5×10 ^6^ cells/ml. Error bar=SEM.

**Supplemental Figure S5.**
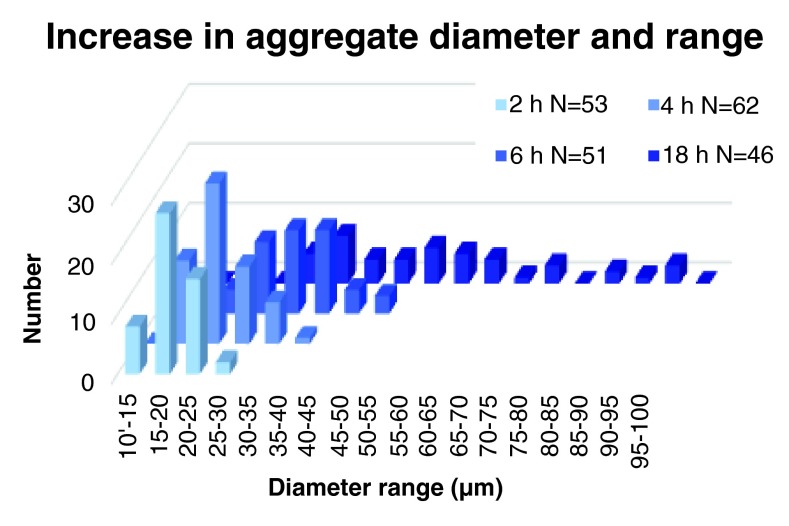
HNK-1 ^+ve^ ENS cells aggregate progressively. Aggregate diameter increased on average and the range of diameters increased over the period 2 h to 18 h in rotation aggregation assays. Starting cell density was 0.3×10 ^6^ cells/ml.

The earliest stage of aggregation for HNK-1 ^+ve^ E8 quail (and E9 chick) midgut ENS cells was as small clumps and strings, at about 2 h in rotating culture. They formed spheres by 4 h, and initially cells bulged from the surface of spheres (resembling a “bunch of grapes”) but the spheres became more smooth-surfaced, and maintained this from 18 h to 48 h ( Figure 4A–D). The diameter of the aggregates increased between 4 h and 48 h ( Figure 4D, Figure 5A) and the range of diameters recorded became wider ( Supplemental Figure S5) but aggregation into a few huge cellular masses did not occur. ENS cells from quail and chick behaved identically in these assays ( Figure 5B).

The spherical form of the aggregates suggests isotropic adhesion forces while the evolution of the aggregates from a “bunch of grapes” appearance to smooth-surfaced spheres indicates an increase in cell-cell adhesion strength *in vitro* with time after initial cell-cell adhesion. A time-dependent increase in adhesive bond energy has been observed in direct measurement of cadherin-mediated adhesion maturation in biophysical tests ( Chu *et al.*, 2004).

**Figure 6.**
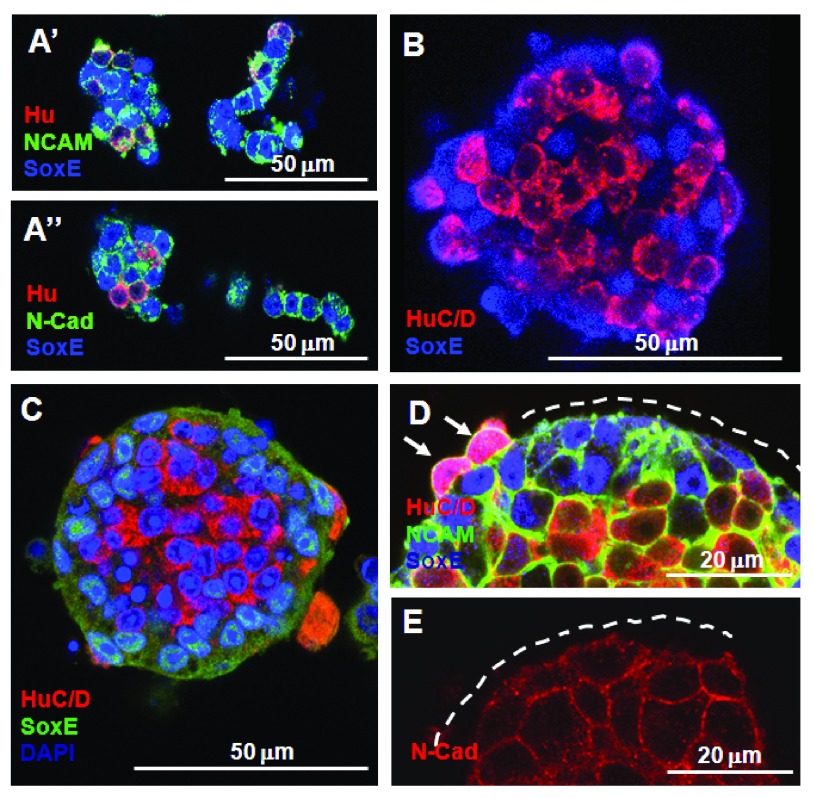
Formation of aggregates by ENS cells in cell-cell adhesion assays correlates with adhesion molecule expression pattern. **A’ A’’**. At 2 h QE8 N-cadherin and NCAM ^+ve^ ENS cells formed small aggregates (mixed Hu ^+ve^ neurons and SoxE ^+ve^ ENC cells) and chains (mostly ENC cells). **B**. At 6 h spherical aggregates were formed but neurons (HuC/D ^+ve^) and ENC cells (SoxE ^+ve^) were only partially segregated. **C**. At 18 h most HuC/D ^+ve^ neurons formed the centre of each spherical aggregate surrounded by SoxE ^+ve^ ENC cells. **D**. NCAM immunoreactivity was low on the external face of SoxE ^+ve^ ENC cells (indicated by dotted line), but high around HuC/D ^+ve^ neurons, whether these were located centrally or on the periphery (arrows). **E**. N-cadherin immunoreactivity was associated with all cells in the aggregate, but was less distinct on the external surface (indicated by dotted line).

SoxE ^+ve^/Hu ^-ve^ (ENC cells) and SoxE ^-ve^/Hu ^+ve^ cells (neurons) occurred first in tiny clumps, with SoxE ^+ve^/Hu ^-ve^ cells predominating in the strings; these ENC cells displayed both N-cadherin and NCAM immunoreactivity ( Figure 6A’, A”). The transient presence of cell-strings has also been described in cells with only cadherin adhesive mechanisms operative ( Takeichi, 1977). Cadherins move on the plane of the membrane and cluster in *cis*, via intercadherin bonds extracellularly and via binding to the cytoskeleton intracellularly ( Hong *et al.*, 2013). The generation of cell strings by SoxE ^+ve^ ENS cells suggests that the ENC cells have a limited number of adhesive molecules on their surface, this *cis*-clustering may restrict cadherin to a few patches capable of mediating adhesion in *trans*.

Raw data for Figure 5A and Supplemental Figure S5 Aggregate size from 2h to 48h ( Rollo *et al.*, 2015c).

Raw data for Figure 5B Aggregate diameters from QE8, ChE9 and mixed populations (Q+Ch) at 18h *in vitro* ( Rollo *et al.*, 2015d).

### ENS cell aggregation is insensitive to fluid shear but sensitive to initial cell density

Varying the speed of rotation (0 and 75 rpm) had little effect on aggregate form or size at 18 h, while aggregates at 150 rpm were somewhat larger in diameter ( Figure 4F–H, Figure 5C). Since increasing rotation rates did not prevent aggregation, the intercellular cell-cell adhesions of these ENS cells must display a rapid “catch” to initiate adhesion, with initial adhesion strength sufficiently high to resist external distractive forces in the highest shear used here. On the other hand, the similar size and shape of the aggregates even down to zero rpm ( Figure 4F, Figure 5C) suggests an overriding intrinsic adhesive mechanism that regulates not only accumulation of cells into aggregates but also governs the preferred size of the aggregates under these conditions.

Varying the initial density of ENS cells in suspension over a 6-fold range (0.167×10 ^6^ to 1.0×10 ^6^ cells/ml) led, counter-intuitively, to smaller aggregates in much larger numbers at higher starting cell densities ( Figure 4I–L, Figure 5D).

Raw data for Figure 5C Aggregate diameter for QE8 cells at 0, 75 and 150 rpm at 18h *in vitro* ( Rollo *et al.*, 2015e).

Raw data for Figure 5D Starting cell density vs aggregate diameter at 18h *in vitro* ( Rollo *et al.*, 2015f).

### The spatial patterning within aggregates indicates ENS neuron cohesion is greater than ENC cell cohesion

Confocal examination of 4–6 h spherical aggregates showed mixed Sox10 ^+ve^ and Hu ^+ve^ cells ( Figure 6B), but by 18 h cells in the aggregates showed most of the Hu ^+ve^ cells located in the centre, with the SoxE ^+ve^ cells forming the outer part of the spheres ( Figure 6C). Labelling for NCAM showed that this adhesion molecule was most strongly expressed on the internal Hu ^+ve^ cells with lower immunoreactivity on the SoxE ^+ve^ cells, and especially low on the outer surface ( Figure 6D). Such differential labelling was also present with labelling for N-cadherin ( Figure 6E).

Confocal examination of four aggregates (diameter range: 63.8–98.7 μm; cell number range 276–861) revealed a remarkably uniform average cell density of 0.24 ± 0.02 cells/(10 μm) ^3^. Likewise the neuron/ENC cell ratio of 1.19 ± 0.06 (Hu ^+ve^/SoxE ^+ve^ cells) was identical to that in the ENS *in vivo* ( Figure 1). The spatial order of central neurons and peripheral ENC cells in the aggregates *in vitro* strikingly resembled that in the ENS ganglia *in vivo* ( Figure 2C) ( Hackett-Jones *et al.*, 2011).

In assays of this kind, cells move within aggregates (“sort out”) with their final equilibrium positions determined by the most favoured adhesive balance, that is, with the least surface free energy of adhesion ( Foty & Steinberg, 2005). To satisfy this, the external position of the ENC cells relative to neurons indicates that the ENC cells must have lower overall adhesive capacity than the neurons, and the lower levels of NCAM immunoreactivity in SoxE ^+ve^ cells is in accord with this. Cell variants with a step difference in adhesiveness segregate especially rapidly in co-aggregates ( Zhang *et al.*, 2011), and this is likely to be represented by the two ENS cell types -neurons and ENC cells- used here.

### ENS cells are mobile within aggregates, allowing sorting out

*In vivo* the neurons form recognisable groups first ( Figure 2B), at a time when the ENC cells still appear randomly placed, and the ENC cells co-assemble around neurons later ( Fairman *et al.*, 1995; Hackett-Jones *et al.*, 2011). In contrast, the final internal/external distribution observed here would be attained from any starting distribution of the cell types ( Steinberg, 2007). Indeed, when we followed aggregation *in vitro*, the neurons and ENC cells were initially mixed ( Figure 6B) and only later did the relative positions of neurons and ENC cells develop ( Figure 6C).

**Figure 7.**
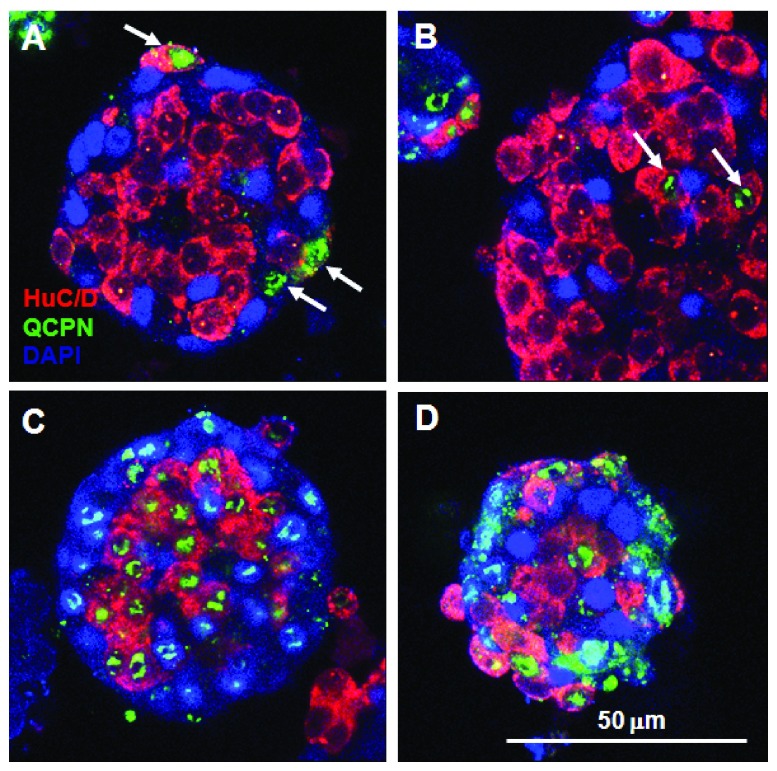
Cells in ENS aggregates are mobile and the surface of formed ENS cell aggregates is relatively non-adhesive. **A**. Few freshly dissociated E8 quail ENS cells (arrows, labelled QCPN ^+ve^ in green) at 3 h *in vitro* attached to the periphery of pre-formed (21 h) E9 chick ENS QCPN ^-ve^ cell aggregates. **B**. At 18 h *in vitro* some quail ENS cells (arrows) relocated deep into the chick ENS 36 h aggregate. **C**. Most quail ENS cells formed entirely quail cell aggregates after 18 h, rather than adhering to pre-formed chick ENS cell aggregates. **D**. QE8 and ChE9 ENS cells when combined as freshly dissociated cells formed mixed aggregates at 6 h, showing that there is no species-related adhesive incompatibility. All specimens were labelled with HuC/D, QCPN and SoxE. Scale bar applies to all images.

To test whether cells were able to sort out within aggregates we combined pre-formed (E9 chick) ENS cell aggregates with freshly dissociated QE8 ENS cells. A few quail cells adhered to the surface of pre-formed ENS cell aggregates when combined for 4 h in rotating culture ( Figure 7A. By 18 h some of these quail ENS cells had penetrated deep into the chick ENS cell aggregates ( Figure 7B). This confirms that cells can move within the aggregates. This is consistent with the SoxE ^+ve^/Hu ^-ve^ ENC cells and SoxE ^-ve^/Hu ^+ve^ neurons physically sorting out, although it does not preclude an additional spatial differentiation whereby internally placed ENC cells differentiate mostly into neurons.

### The external surface of ENS cell aggregates is poorly adhesive for ENS cells

When freshly dissociated QE8 HNK-1 ^+ve^ cells were added to pre-formed (18 h) chick E9 ENS cell aggregates, QCPN-labelling showed that only a few quail cells adhered to the outer surface of pre-formed chick cell aggregates ( Figure 7A), and most quail ENS cells formed separate aggregates entirely of quail cells ( Figure 7C). This separation was not caused by species-specific adhesive differences, because when freshly dissociated E9 chick and QE8 ENS cells were mixed at the time of dissociation, all cell aggregates were a mixture of both chick and quail cells ( Figures 5B, Figure 7D).

This shows that the outer surface of pre-formed aggregates is relatively non-adhesive, and is consistent with the observation that the outer surface of the peripheral cells of aggregates showed low immunoreactivity for CAMs ( Figures 6D, E). We therefore propose that the outer SoxE ^+ve^ ENC cells segregate a limited number of CAMs mainly to the internal face to bind to similar but more numerous molecules on the neurons, leaving the external surface relatively non-adhesive. This process would automatically restrict the size of ENS cell aggregates by preventing new cells from binding to the surface.

### Altering the ENC cell/neuron ratio alters the aggregate size

**Figure 8.**
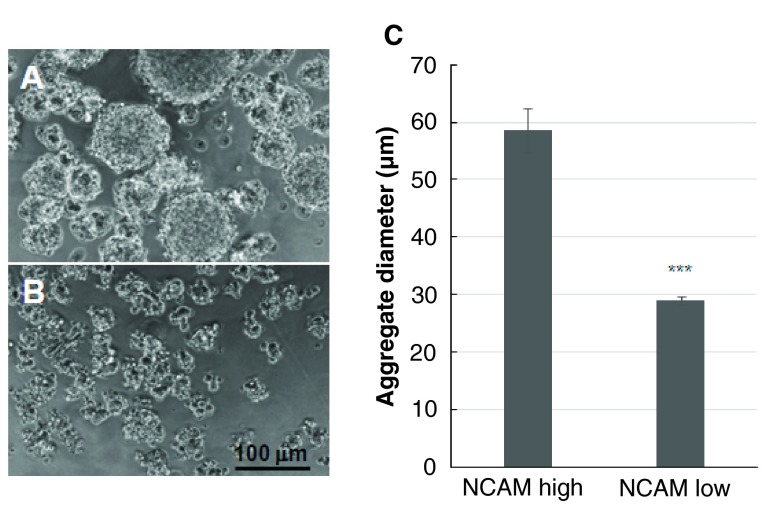
HNK-1 ^+ve^ QE8 ENS cell aggregate size is influenced by NCAM levels. **A**. NCAM-high FACS fraction of HNK-1 ^+ve^ ENS cells form aggregates with a wide range of diameters including very large aggregates. **B**. NCAM-low fraction ENS cells form small misshapen aggregates. **C**. Histogram of aggregate diameters formed at 18h *in vitro* by NCAM-high and NCAM-low ENS cell fractions. Difference is highly significant (*** p<0.001). Starting cell density 0.67 ×10 ^6^ cells/ml. Error bar=SEM.

If the peripherally located Sox10 ^+ve^ ENC cells in the aggregates form an insulating coating preventing ever-larger aggregates from forming, then reducing the number of these cells should allow larger aggregates to form. By combining NCAM with HNK-1 FACS, we produced HNK-1 ^+ve^ populations of higher and lower NCAM levels ( Figure 6D, Supplemental Figure S2C). Previous immunolabelling *in vivo* ( Hackett-Jones *et al.*, 2011) and here in aggregates *in vitro* shows that the NCAM-high sub-population will be enriched for neurons, and the NCAM-low sub-population depleted in neurons. Culturing these cells in Terasaki wells confirmed this was the case, both populations had SoxE ^+ve^ cells but HuC/D ^+ve^ cells with E-C8 ^+ve^, Tuj1 ^+ve^ neurites occurred only in the NCAM high fraction. Aggregation of NCAM-high and NCAM-low sub-populations produced respectively larger and smaller irregularly shaped aggregates than was usual for unsorted HNK-1 ^+ve^ cells ( Figure 8A–C).

Raw data for Figure 8C Comparison of aggregate size in NCAM-high and NCAM-low fractions as sorted by FACS ( Rollo *et al.*, 2015g).

### Different NC cell ganglia show aggregation differences

**Figure 9.**
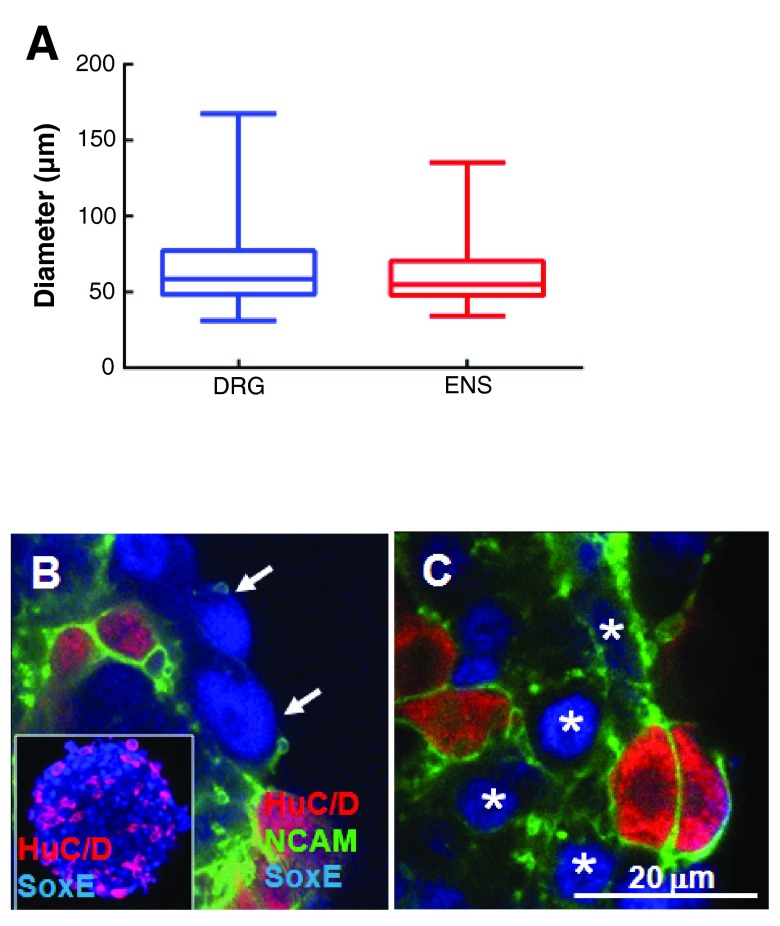
QE8 DRG cell aggregates *in vitro* have different internal structure than ENS cell aggregates. **A**. QE8 DRG and ENS HNK-1 ^+ve^ cell aggregates were similar in average size and size distribution. Box plots show the first quartile to inter quartile range and whiskers show minimum and maximum range of data, while the median is represented by a vertical line. There was no difference between DRG and ENS in aggregate size when analysed using Mann-Whitney test for nonparametric data. Starting cell density was 0.5 ×10 ^6^ cells/ml. **B**. Unlike QE8 ENS cell aggregates, Hu ^+ve^ neurons and SoxE ^+ve^ cells are not segregated in DRG aggregates (inset), but like ENS aggregates, peripheral SoxE ^+ve^ cells show little outer NCAM labelling (arrows). **C**. In contrast, internal SoxE ^+ve^ cells (stars) as well as surface and internal Hu ^+ve^ cells show strong NCAM labelling in DRG cell aggregates.

Quail E8 trunk DRG were dissociated as for the ENS cells and placed in aggregation assays. Like the ENS cells, DRG cells rapidly formed aggregates with similar average size although the range of size of DRG aggregates was larger ( Figure 9A). Interestingly, the internal structure of the aggregates was strikingly different: SoxE ^+^/Hu ^-ve^ cells and SoxE ^-^/Hu ^+ve^ neurons were mixed not segregated ( Figure 9B inset). In addition, although all Hu+ve neurons were strongly NCAM ^+ve^ and all outer SoxE ^+ve^ cell membranes were deficient in NCAM labelling (as in equivalent ENS cells), the internal SoxE ^+ve^ cells were strongly NCAM-labelled ( Figure 9B, C).

*In vivo*, NC-derived cells coalesce to form DRG as early as about E4 (in chick; equivalent to E3.5 in quail), and at these early stages the DRG display NC cell/neuron segregation as seen for ENS cells, with all neurons placed centrally ( Wakamatsu *et al.*, 2000). As early as E5 in chick, however, early DRG glial cells identified by transitin expression (which are also SoxE ^+ve^) spread centrally between the DRG neurons ( Henion *et al.*, 2000). We propose that the different sorting behavior of QE8 ENS cells and QE8 DRG cells represents a difference in developmental stage of the two ganglion types of the same chronological age, with DRG being much further advanced in ganglion cell differentiation, marked by the appearance of highly NCAM ^+ve^ glial cells. The morphogenetic consequence of this drives the internal relocalisation of glial cells among the neurons they support by E8 in trunk DRG. We propose that a similar process evolves later, by E14, in midgut ENS ( Figure 2D).

Raw data for Figure 9A Aggregate size for QE8 DRG and ENS HNK-1 ^+ve^ cells ( Rollo *et al.*, 2015h).

Raw data for ENC: Neuron ratio (page 12) EN cell: neuron ratio and aggregate size ( Rollo *et al.*, 2015i).

### Conclusions: intrinsic regulation of cell distribution and size of ENS ganglia

**Figure 10.**
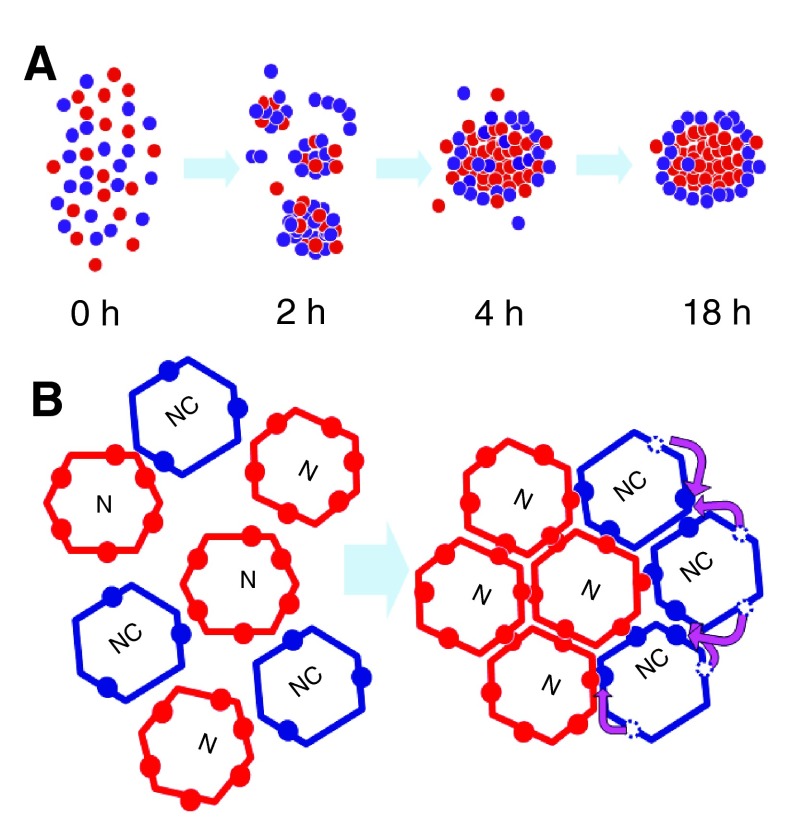
Scheme of ENS neuron (red) and ENC (blue) aggregation. **A**. Initially ENC cells and neurons cohere in a disorderly fashion (2–4 h) but gradually the neurons sort out to a central position (18 h), indicating neurons have a greater adhesive bond energy. **B**. Neurons and ENC cells have adhesive molecules (coloured circles) which are free to move in the cell surface, but the neurons have more adhesive molecules. Cell-cell adhesion requires juxtaposition in *trans* of two adhesive molecules (red-red, blue-blue or red-blue). To minimise adhesive free energy, adhesive molecules on ENC cells move (pink arrows) in order to form an adhesion, thereby denuding the outer face of adhesion molecules. This insulates the group from further adhesion.

A mechanism of separation by sorting of more and less adhesive cells would produce the observed core/shell spatial distribution of SoxE ^+ve^ and Hu ^+ve^ cells ( Figure 10A). Translocation of a numerically limited number of CAMs in the plane of the cell membrane to the internal-facing side of the less adhesive cells (i.e. the Sox10 ^+ve^ cells) to engage the more numerous homophilic CAMs on the more adhesive cells (i.e. Hu ^+ve^ neurons) ( Figure 10B) would automatically limit the size of the aggregates by denuding the external surface of CAMs. This mechanism would predict that the size of the aggregate depends at least in part on the ratio of more adhesive neurons to less adhesive ENC cells. We suggest that this operates also *in vivo* and is of importance for ensuring that the ENS consists of relatively small and uniform ganglia. However, alteration of adhesion of ENS cells to ECM leads to ENS ganglia of abnormal size, shape and pattern of distribution *in vivo* ( Breau *et al.*, 2009; Broders-Bondon *et al.*, 2012). This shows that the balance of cell-ECM adhesion as well as cell-cell adhesion, needs to be taken into account to achieve correct ENS ganglionic morphogenesis.

### Data availability

*F1000Research: Dataset 1. Raw data for Figure 1, 10.5256/f1000research.6370.d45928*

*F1000Research: Dataset 2. Raw data for Figure 3a, b and c, 10.5256/f1000research.6370.d45929*

*F1000Research: Dataset 3. Raw data for Figure 5a and Supplemental Figure S5, 10.5256/f1000research.6370.d45930*

*F1000Research: Dataset 4. Raw data for Figure 5b, 10.5256/f1000research.6370.d45931*

*F1000Research: Dataset 5. Raw data for Figure 5c, 10.5256/f1000research.6370.d45932*

*F1000Research: Dataset 6. Raw data for Figure 5d, 10.5256/f1000research.6370.d45933*

*F1000Research: Dataset 7. Raw data for Figure 8c, 10.5256/f1000research.6370.d45934*

*F1000Research: Dataset 8. Raw data for Figure 9a, 10.5256/f1000research.6370.d45935*

*F1000Research: Dataset 9. Raw data for Neuron: ENC ratio (page 12), 10.5256/f1000research.6370.d45939*

*F1000Research: Dataset 10. Raw data for Supplemental Figure S3, 10.5256/f1000research.6370.d45936*

## Figures and Tables

**Supplementary Table T1.** Antibodies and fluorescent probes.

Antibody or probe to:	Species and type	Dil ^n^	Source, code
BrdU (bromodeoxy uridine)	Mouse IgG	1/100	Amersham BU-1
DNA (nuclei)	DAPI	0.5 μM	LifeTechnol.
HNK-1 (3-sulphoglucuronosyl antigen)	Mouse IgM	1/20	MCRI
HuC/D (neuronal Elav family)	Mouse IgG	1/200	Molecular Probes 16A11
HU (neuronal Elav family)	Human IgG	1/6000	Dr. Vanda Lennon, Mayo Clinic, MN
NAPA-73 (neurofil.-assoc. prot., 73 kDa)	Mouse IgM	1/20	DSHB E/C8
N-cadherin	Mouse IgG	1/100	Sigma GC-4
N-cadherin	Rabbit IgG	1/200	Abcam ab18203
NCAM	Rabbit IgG	1/200	Chemicon AB5032
NCAM extracellular	Mouse IgG	1/100	DSHB 5e
Neurofilament M	Rabbit IgG	1/100	Chemicon AB1987
QCPN (quail not chick perinuclear antigen)	Mouse IgG	1/50	DSHB
SMA (smooth muscle actin)	Mouse IgG	1/200	Sigma 1A4
SoxE (Sox-8,9 and 10)	Rabbit IgG	1/2500	Dr Craig Smith, MCRI
TuJ-1 (neuron-specific β-III tubulin)	Mouse IgG	1/400	R&D, MAB1195
Phosphohistone-H3	Rabbit IgG	1/200	Upstate #07-081
**Detection probes:**
mouse IgGγ: FITC	Goat IgG	1/50	Jackson Immunores. 115-545-071
mouse IgG: Alexa 350	Goat IgG	1/100	Molecular Probes MP A21049
mouse IgG: Alexa 488	Goat IgG	1/400	Molecular Probes MP A11029
mouse IgG: Alexa 594	Donkey IgG	1/2000	Molecular Probes MP A21203
mouse IgMμ: Texas Red	Goat IgG	1/400	Jackson Immunores. 115-075-075
mouse IgG: Alexa 647	Goat IgG	1/1000	Molecular Probes MP A21235
rat IgG: biotin	Donkey IgG	1/100	Jackson Immunores. 712 065 153
rabbit IgG: Alexa 488	Goat IgG	1/400	Molecular Probes MP A110334
rabbit IgG: Alexa 594	Donkey IgG	1/2000	Molecular Probes MP A-21207
rabbit IgG: biotin	Donkey IgG	1/100	Jackson Immunores. 711 065 152
Biotin (Streptavidin: AMCA)	NA	1/500	Jackson Immunores. 016 150 084

## Supplementary information

Raw data for Supplemental Figure S3 Full qPCR analysis of HNK-1 sorted cells ( Rollo *et al.*, 2015j).

## References

[ref-1] Allan IJ Newgreen DF: The origin and differentiation of enteric neurons of the intestine of the fowl embryo. *Am J Anat.* 1980;157(2):137–154. 10.1002/aja.1001570203 7405865

[ref-2] Barde YA: Neurotrophins: a family of proteins supporting the survival of neurons. *Prog Clin Biol Res.* 1994;390:45–56. 7724649

[ref-3] Binder BJ Landman KA Simpson MJ: Modeling proliferative tissue growth: a general approach and an avian case study. *Phys Rev E Stat Nonlin Soft Matter Phys.* 2008;78(3 Pt 1):031912. 10.1103/PhysRevE.78.031912 18851070

[ref-4] Breau MA Dahmani A Broders-Bondon F: Beta1 integrins are required for the invasion of the caecum and proximal hindgut by enteric neural crest cells. *Development.* 2009;136(16):2791–2801. 10.1242/dev.031419 19633172

[ref-5] Breau MA Pietri T Eder O: Lack of beta1 integrins in enteric neural crest cells leads to a Hirschsprung-like phenotype. *Development.* 2006;133(9):1725–1734. 10.1242/dev.02346 16571628

[ref-6] Broders-Bondon F Paul-Gilloteaux P Carlier C: N-cadherin and β1-integrins cooperate during the development of the enteric nervous system. *Dev Biol.* 2012;364(2):178–191. 10.1016/j.ydbio.2012.02.001 22342243

[ref-7] Chalazonitis A D'Autreaux F Guha U: Bone morphogenetic protein-2 and -4 limit the number of enteric neurons but promote development of a TrkC-expressing neurotrophin-3-dependent subset. *J Neurosci.* 2004;24(17):4266–4282. 10.1523/JNEUROSCI.3688-03.2004 15115823 PMC6729284

[ref-8] Chalazonitis A D'Autreaux F Pham TD: Bone morphogenetic proteins regulate enteric gliogenesis by modulating ErbB3 signaling. *Dev Biol.* 2011;350(1):64–79. 10.1016/j.ydbio.2010.11.017 21094638 PMC3034360

[ref-9] Chalazonitis A Gershon MD Greene LA: Cell death and the developing enteric nervous system. *Neurochem Int.* 2012;61(6):839–847. 10.1016/j.neuint.2012.01.028 22342822 PMC3398212

[ref-10] Chu YS Thomas WA Eder O: Force measurements in E-cadherin-mediated cell doublets reveal rapid adhesion strengthened by actin cytoskeleton remodeling through Rac and Cdc42. *J Cell Biol.* 2004;167(6):1183–1194. 10.1083/jcb.200403043 15596540 PMC2172605

[ref-11] Conner PJ Focke PJ Noden DM: Appearance of neurons and glia with respect to the wavefront during colonization of the avian gut by neural crest cells. *Dev Dyn.* 2003;226(1):91–98. 10.1002/dvdy.10219 12508228

[ref-12] D'Autreaux F Morikawa Y Cserjesi P: *Hand2* is necessary for terminal differentiation of enteric neurons from crest-derived precursors but not for their migration into the gut or for formation of glia. *Development.* 2007;134(12):2237–2249. 10.1242/dev.003814 17507395

[ref-13] Delalande JM Natarajan D Vernay B: Vascularisation is not necessary for gut colonisation by enteric neural crest cells. *Dev Biol.* 2014;385(2):220–229. 10.1016/j.ydbio.2013.11.007 24262984 PMC3928993

[ref-14] Druckenbrod NR Epstein ML: The pattern of neural crest advance in the cecum and colon. *Dev Biol.* 2005;287(1):125–133. 10.1016/j.ydbio.2005.08.040 16197939

[ref-15] Epstein ML Mikawa T Brown AM: Mapping the origin of the avian enteric nervous system with a retroviral marker. *Dev Dyn.* 1994;201(3):236–244. 10.1002/aja.1002010307 7881127

[ref-16] Epstein ML Poulsen KT Thiboldeaux R: Formation of ganglia in the gut of the chick embryo. *J Comp Neurol.* 1991;307(2):189–199. 10.1002/cne.903070203 1856323

[ref-17] Fairman CL Clagett-Dame M Lennon VA: Appearance of neurons in the developing chick gut. *Dev Dyn.* 1995;204(2):192–201. 10.1002/aja.1002040210 8589443

[ref-18] Faure C Chalazonitis A Rheaume C: Gangliogenesis in the enteric nervous system: roles of the polysialylation of the neural cell adhesion molecule and its regulation by bone morphogenetic protein-4. *Dev Dyn.* 2007;236(1):44–59. 10.1002/dvdy.20943 16958105

[ref-19] Flynn B Bergner AJ Turner KN: Effect of *Gdnf* haploinsufficiency on rate of migration and number of enteric neural crest-derived cells. *Dev Dyn.* 2007;236(1):134–141. 10.1002/dvdy.21013 17103416

[ref-20] Foty RA Steinberg MS: The differential adhesion hypothesis: a direct evaluation. *Dev Biol.* 2005;278(1):255–263. 10.1016/j.ydbio.2004.11.012 15649477

[ref-21] Furness JB: The enteric nervous system and neurogastroenterology. *Nat Rev Gastroenterol Hepatol.* 2012;9(5):286–294. 10.1038/nrgastro.2012.32 22392290

[ref-22] Goldstein AM Brewer KC Doyle AM: BMP signaling is necessary for neural crest cell migration and ganglion formation in the enteric nervous system. *Mech Dev.* 2005;122(6):821–833. 10.1016/j.mod.2005.03.003 15905074

[ref-23] Hackett-Jones EJ Landman KA Newgreen DF: On the role of differential adhesion in gangliogenesis in the enteric nervous system. *J Theor Biol.* 2011;287:148–159. 10.1016/j.jtbi.2011.07.013 21816161

[ref-24] Hamburger V Hamilton HL: A series of normal stages in the development of the chick embryo. *J Morphol.* 1951;88(1):49–92. 10.1002/jmor.1050880104 24539719

[ref-25] Hao MM Anderson RB Kobayashi K: The migratory behavior of immature enteric neurons. *Dev Neurobiol.* 2009;69(1):22–35. 10.1002/dneu.20683 18985707

[ref-26] Hatch J Mukouyama YS: Spatiotemporal mapping of vascularization and innervation in the fetal murine intestine. *Dev Dyn.* 2015;244(1):56–68. 10.1002/dvdy.24178 25138596 PMC8538805

[ref-27] Hearn CJ Murphy M Newgreen D: GDNF and ET-3 differentially modulate the numbers of avian enteric neural crest cells and enteric neurons *in vitro*. *Dev Biol.* 1998;197(1):93–105. 10.1006/dbio.1998.8876 9578621

[ref-28] Henion PD Blyss GK Luo R: Avian transitin expression mirrors glial cell fate restrictions during neural crest development. *Dev Dyn.* 2000;218(1):150–159. 10.1002/(SICI)1097-0177(200005)218:1<150::AID-DVDY13>3.0.CO;2-6 10822267

[ref-29] Hong S Troyanovsky RB Troyanovsky SM: Binding to F-actin guides cadherin cluster assembly, stability, and movement. *J Cell Biol.* 2013;201(1):131–143. 10.1083/jcb.201211054 23547031 PMC3613698

[ref-30] Jiang Y Liu MT Gershon MD: Netrins and DCC in the guidance of migrating neural crest-derived cells in the developing bowel and pancreas. *Dev Biol.* 2003;258(2):364–384. 10.1016/S0012-1606(03)00136-2 12798294

[ref-31] Kalcheim C Barde YA Thoenen H: *In vivo* effect of brain-derived neurotrophic factor on the survival of developing dorsal root ganglion cells. *Embo J.* 1987;6(10):2871–2873. 3691474 10.1002/j.1460-2075.1987.tb02589.x PMC553720

[ref-32] Kasemeier-Kulesa JC Bradley R Pasquale EB: Eph/ephrins and N-cadherin coordinate to control the pattern of sympathetic ganglia. *Development.* 2006;133(24):4839–4847. 10.1242/dev.02662 17108003

[ref-33] Kasemeier-Kulesa JC McLennan R Romine MH: CXCR4 controls ventral migration of sympathetic precursor cells. *J Neurosci.* 2010;30(39):13078–13088. 10.1523/JNEUROSCI.0892-10.2010 20881125 PMC6633505

[ref-34] Lei J Howard MJ: Targeted deletion of Hand2 in enteric neural precursor cells affects its functions in neurogenesis, neurotransmitter specification and gangliogenesis, causing functional aganglionosis. *Development.* 2011;138(21):4789–4800. 10.1242/dev.060053 21989918 PMC3190387

[ref-35] Mwizerwa O Das P Nagy N: Gdnf is mitogenic, neurotrophic, and chemoattractive to enteric neural crest cells in the embryonic colon. *Dev Dyn.* 2011;240(6):1402–1411. 10.1002/dvdy.22630 21465624 PMC3092856

[ref-36] Nagy N Burns AJ Goldstein AM: Immunophenotypic characterization of enteric neural crest cells in the developing avian colorectum. *Dev Dyn.* 2012;241(5):842–851. 10.1002/dvdy.23767 22411589 PMC3428738

[ref-37] Nagy N Goldstein AM: Endothelin-3 regulates neural crest cell proliferation and differentiation in the hindgut enteric nervous system. *Dev Biol.* 2006;293(1):203–217. 10.1016/j.ydbio.2006.01.032 16519884

[ref-38] Nagy N Mwizerwa O Yaniv K: Endothelial cells promote migration and proliferation of enteric neural crest cells via beta1 integrin signaling. *Dev Biol.* 2009;330(2):263–272. 10.1016/j.ydbio.2009.03.025 19345201 PMC2690696

[ref-39] Newgreen DF Hartley L: Extracellular matrix and adhesive molecules in the early development of the gut and its innervation in normal and *spotting lethal* rat embryos. *Acta Anat (Basel).* 1995;154(4):243–260. 10.1159/000147776 8773711

[ref-40] Okamura Y Saga Y: Notch signaling is required for the maintenance of enteric neural crest progenitors. *Development.* 2008;135(21):3555–3565. 10.1242/dev.022319 18832397

[ref-41] Peterson AJ Menheniott TR O'Connor L: *Helicobacter pylori* infection promotes methylation and silencing of *trefoil* factor 2, leading to gastric tumor development in mice and humans. *Gastroenterology.* 2010;139(6):2005–2017. 10.1053/j.gastro.2010.08.043 20801119 PMC3970568

[ref-42] Rollo BN Zhang D Simkin JE: Dataset 1 in: Why are enteric ganglia so small? Role of differential adhesion of enteric neurons and enteric neural crest cells. *F1000Research.* 2015a Data Source 10.12688/f1000research.6370.1 PMC4448751 26064478

[ref-43] Rollo BN Zhang D Simkin JE: Dataset 2 in: Why are enteric ganglia so small? Role of differential adhesion of enteric neurons and enteric neural crest cells. *F1000Research.* 2015b Data Source 10.12688/f1000research.6370.1 PMC4448751 26064478

[ref-44] Rollo BN Zhang D Simkin JE: Dataset 3 in: Why are enteric ganglia so small? Role of differential adhesion of enteric neurons and enteric neural crest cells. *F1000Research.* 2015c Data Source 10.12688/f1000research.6370.1 PMC4448751 26064478

[ref-45] Rollo BN Zhang D Simkin JE: Dataset 4 in: Why are enteric ganglia so small? Role of differential adhesion of enteric neurons and enteric neural crest cells. *F1000Research.* 2015d Data Source 10.12688/f1000research.6370.1 PMC4448751 26064478

[ref-46] Rollo BN Zhang D Simkin JE: Dataset 5 in: Why are enteric ganglia so small? Role of differential adhesion of enteric neurons and enteric neural crest cells. *F1000Research.* 2015e Data Source 10.12688/f1000research.6370.1 PMC4448751 26064478

[ref-47] Rollo BN Zhang D Simkin JE: Dataset 6 in: Why are enteric ganglia so small? Role of differential adhesion of enteric neurons and enteric neural crest cells. *F1000Research.* 2015f Data Source 10.12688/f1000research.6370.1 PMC4448751 26064478

[ref-48] Rollo BN Zhang D Simkin JE: Dataset 7 in: Why are enteric ganglia so small? Role of differential adhesion of enteric neurons and enteric neural crest cells. *F1000Research.* 2015g Data Source 10.12688/f1000research.6370.1 PMC4448751 26064478

[ref-49] Rollo BN Zhang D Simkin JE: Dataset 8 in: Why are enteric ganglia so small? Role of differential adhesion of enteric neurons and enteric neural crest cells. *F1000Research.* 2015h Data Source 10.12688/f1000research.6370.1 PMC4448751 26064478

[ref-50] Rollo BN Zhang D Simkin JE: Dataset 9 in: Why are enteric ganglia so small? Role of differential adhesion of enteric neurons and enteric neural crest cells. *F1000Research.* 2015i Data Source 10.12688/f1000research.6370.1 PMC4448751 26064478

[ref-51] Rollo BN Zhang D Simkin JE: Dataset 10 in: Why are enteric ganglia so small? Role of differential adhesion of enteric neurons and enteric neural crest cells. *F1000Research.* 2015j Data Source 10.12688/f1000research.6370.1 PMC4448751 26064478

[ref-52] Schrenk S Schuster A Klotz M: Vascular and neural stem cells in the gut: do they need each other? *Histochem Cell Biol.* 2015;143(4):397–410. 10.1007/s00418-014-1288-9 25371326

[ref-53] Simkin JE Zhang D Rollo BN: Retinoic Acid upregulates ret and induces chain migration and population expansion in vagal neural crest cells to colonise the embryonic gut. *PLoS One.* 2013;8(5):e64077. 10.1371/journal.pone.0064077 23717535 PMC3661488

[ref-54] Simpson MJ Zhang DC Mariani M: Cell proliferation drives neural crest cell invasion of the intestine. *Dev Biol.* 2007;302(2):553–568. 10.1016/j.ydbio.2006.10.017 17178116

[ref-55] Steinberg MS: Differential adhesion in morphogenesis: a modern view. *Curr Opin Genet Dev.* 2007;17(4):281–286. 10.1016/j.gde.2007.05.002 17624758

[ref-56] Steinberg MS Armstrong PB Granger RE: On the recovery of adhesiveness by trypsin-dissociated cells. *J Membr Biol.* 1973;13(2):97–128. 10.1007/BF01868223 4205052

[ref-57] Takahashi M Iwashita T Santoro M: Co-segregation of MEN2 and Hirschsprung’s disease: the same mutation of RET with both gain and loss-of-function? *Hum Mutat.* 1999;13(4):331–336. 10.1002/(SICI)1098-1004(1999)13:4<331::AID-HUMU11>3.0.CO;2-%23 10220148

[ref-58] Takeichi M: Functional correlation between cell adhesive properties and some cell surface proteins. *J Cell Biol.* 1977;75(2 Pt 1):464–474. 10.1083/jcb.75.2.464 264120 PMC2109947

[ref-59] Teillet MA Kalcheim C Le Douarin NM: Formation of the dorsal root ganglia in the avian embryo: segmental origin and migratory behavior of neural crest progenitor cells. *Dev Biol.* 1987;120(2):329–347. 10.1016/0012-1606(87)90236-3 3549390

[ref-60] Theocharatos S Wilkinson DJ Darling S: Regulation of progenitor cell proliferation and neuronal differentiation in enteric nervous system neurospheres. *PLoS One.* 2013;8(1):e54809. 10.1371/journal.pone.0054809 23372773 PMC3553067

[ref-61] Tsarovina K Schellenberger J Schneider C: Progenitor cell maintenance and neurogenesis in sympathetic ganglia involves Notch signaling. *Mol Cell Neurosci.* 2008;37(1):20–31. 10.1016/j.mcn.2007.08.010 17920293

[ref-62] Wakamatsu Y Maynard TM Weston JA: Fate determination of neural crest cells by NOTCH-mediated lateral inhibition and asymmetrical cell division during gangliogenesis. *Development.* 2000;127(13):2811–2821. 10851127 10.1242/dev.127.13.2811

[ref-63] Wallace AS Barlow AJ Navaratne L: Inhibition of cell death results in hyperganglionosis: implications for enteric nervous system development. *Neurogastroenterol Motil.* 2009;21(7):768–e49. 10.1111/j.1365-2982.2009.01309.x 19400926

[ref-64] Wu JJ Chen JX Rothman TP: Inhibition of *in vitro* enteric neuronal development by endothelin-3: mediation by endothelin B receptors. *Development.* 1999;126(6):1161–1173. 10021336 10.1242/dev.126.6.1161

[ref-65] Yntema CL Hammond WS: The origin of intrinsic ganglia of trunk viscera from vagal neural crest in the chick embryo. *J Comp Neurol.* 1954;101(2):515–541. 10.1002/cne.901010212 13221667

[ref-66] Young HM Bergner AJ Anderson RB: Dynamics of neural crest-derived cell migration in the embryonic mouse gut. *Dev Biol.* 2004;270(2):455–473. 10.1016/j.ydbio.2004.03.015 15183726

[ref-67] Young HM Bergner AJ Simpson MJ: Colonizing while migrating: how do individual enteric neural crest cells behave? *BMC Biol.* 2014;12:23. 10.1186/1741-7007-12-23 24670214 PMC4101823

[ref-68] Young HM Hearn CJ Farlie PG: GDNF is a chemoattractant for enteric neural cells. *Dev Biol.* 2001;229(2):503–516. 10.1006/dbio.2000.0100 11150245

[ref-69] Zarzosa A Grassme K Tanaka E: Axolotls with an under- or oversupply of neural crest can regulate the sizes of their dorsal root ganglia to normal levels. *Dev Biol.* 2014;394(1):65–82. 10.1016/j.ydbio.2014.08.001 25111151

[ref-70] Zhang D Brinas IM Binder BJ: Neural crest regionalisation for enteric nervous system formation: implications for Hirschsprung's disease and stem cell therapy. *Dev Biol.* 2010;339(2):280–294. 10.1016/j.ydbio.2009.12.014 20083101

[ref-71] Zhang Y Niswander L: Zic2 is required for enteric nervous system development and neurite outgrowth: a mouse model of enteric hyperplasia and dysplasia. *Neurogastroenterol Motil.* 2013;25(6):538–541. 10.1111/nmo.12101 23413832 PMC3862053

[ref-72] Zhang Y Thomas GL Swat M: Computer simulations of cell sorting due to differential adhesion. *PLoS One.* 2011;6(10):e24999. 10.1371/journal.pone.0024999 22028771 PMC3196507

