# Supplementary material for: Why are enteric ganglia so small? Role of differential adhesion of enteric neurons and enteric neural crest cells
Source: F1000Res. 2015 May 12;4:113. [Version 1] doi: 10.12688/f1000research.6370.1 (PMC4448751; doi:10.12688/f1000research.6370.1)
Supplement: Peer review history

# Peer review: Referee response for version 1

**DOI:** 10.5256/f1000research.6830.r8815

**Published:** 2015-05-29

**Authors:** Hans-Henning Epperlein

**Affiliations:** 1 Center for Regenerative Therapies Dresden (CRTD), Technische Universität Dresden, Dresden, Germany

Rollo *et al*. studied ganglion formation of ENS cells in vitro by using disaggregated cells from midguts of E5 and E8 quail and E9 chick embryos. Aggregation was investigated in cell-cell adhesion assays with HNK-1 fluorescence-activated cell sorting (FACS). While the developing neurons (Hu-positive) sorted out to the centre of the aggregates, NC cells (SoxE-positive) stayed more peripherally. This sorting behaviour was consistent with a higher adhesion centrally and a parallely higher N-cadherin- and NCAM-labelling. This main result suggests that a differential adhesion mechanism of enteric neurons and NC cells controls the inner/outer layering of the ganglia with the ENC cell fraction limiting the size of aggregates.

The merit of this careful and detailed study is that it tries to visualize quantitatively at a cellular level what cannot be observed *in vivo *in this way or only exemplarily with severe difficulties via 3D-reconstructions. I recommend indexation. I have a small “major” concern and give a list of several minor comments below.

**Major concern**

Results and discussion p 5, section “ **ENS ganglia undergo progressive morphogenesis *in vivo***”: The size of the aggregates forming *in vivo *(see Fig. 2D) should be indicated here in this section, i.e. as much as one can recognize with the confocal studies used (diameter/cell number). Then this size and the *in vitro *size can be compared. Obviously the *in vitro *aggregates are much larger, similar to DRG size in vivo. This should be explained. The *in vitro *size of aggregates is indicated in detail on p 9. (see also minor comments 4, 8, 12).

**Minor comments**

1. **Introduction**: The authors might consider to move paragraph 4 starting with “The final ENS cell population is enormous…” after paragraph 1.
2. **Introduction, paragraph 3: citation Teillet et al. 87**; here 1-2 papers from R. Mayor could be added (e.g. Theveneau and Mayor, 2012; Kuriyama and Mayor, 2008).
3. **Introduction, paragraph 4** What is “DCC”?
4. **Last paragraph of Introduction**: “In particular we wished to explore why the ENS ganglia are similar and small in size……” If this is your wish you should add here 2-3 sentences about how “real” your results are. How much bigger/smaller are your ganglia obtained *in vitro *compared to those on the slices cut from an *in vivo *specimen (Fig. 2)?  Think also of and compare the *in vitro*-sizes of enteric ganglia to your previous work!
5. **Mat.+Meth.: “**Counts of myenteric ENS cells in wholemounts”. One does not know what “Hu” stands for and why “SoxE” is mentioned here but Sox-10 further down on the page (last line). Explanation of abbreviations here or before the paper starts?
6. **“Cell dissociation”**.  While a non-expert might imagine how a “midgut” is removed, the skill that is needed to harvest DRG may be beyond his/her imagination. Add perhaps 1-2 sentences for explanation?
  “The intestinal tissue pooled from 15–60 embryos”: why 15-60? Is this one of many similar preparations using between 15 and 60 guts? Otherwise you should indicate a fixed number.
  One gets no information about the proportion of ENS cells obtained from the myenteric and submucosal plexuses. Are the ganglia in both places equal in size and structure? At certain developmental stages there could be many more Hu-positive or SoxE-positive ganglia in the myenteric than in the submucosal plexuses because the latter are settled earlier.
7. **Section: “** **Fluorescent labelling and fluorescence-activated cell sorting (FACS)”:** It is hardly imaginable that from such a broth obtained by filtering disaggregated tissue or single cells  through a 30 µm strainer cells were obtained that could form such beautiful aggregates as shown in Fig. 6. May be you can add a sentence about the consistency of the filtered cells?
8. **Results: “**Figure 2”: Why are myenteric plexuses chosen and not submucosal ones? What is the final size of a ganglion in Fig. 2D (in vivo) compared to Fig. 6C ( *in vitro*)? Is the diameter range (63.8-98.7 µm) and the cell number range (276-861) indicated on p. 9 (right column) comparable to the diameters/sizes of the *in vivo *aggregates ?
9. “ **ENS cell clusters *in vitro *are due to cell aggregation, not proliferation”. **This section is nearly too short to merit section status – could it be subsumed somewhere else?
10. **Fig. 6D**: “NCAM” is hardly readable.
11. “ **ENS cells are mobile within aggregates, allowing sorting out”. **I only wonder whether this initial mobility of ENS and ENC cells and their later sorting out and finally the outer placement of the ENC cells with restricting ENS cells is not all a little contradictory?
12. **Fig. 9: ..DRG…..cell aggregates were similar in average size… **But the DRG would be definitely much bigger than the enteric ganglia ? If not, then your *in vitro *aggregates are much bigger than the *in vivo *ones! See again point 8 (above) and compare ENS aggregate sizes in Fig. 2d and Fig. 6C with DRG size in Fig. 9. May be you could discuss that at the very end of the paper?

I have read this submission. I believe that I have an appropriate level of expertise to confirm that it is of an acceptable scientific standard.

# Peer review: Referee response for version 1

**DOI:** 10.5256/f1000research.6830.r8623

**Published:** 2015-05-26

**Authors:** Miles L. Epstein, Amanda Barlow

**Affiliations:** 1 Department of Neuroscience, School of Medicine and Public Health, University of Wisconsin-Madison, Madison, WI, USA; 2 Department of Surgery, University of Wisconsin-Madison, Madison, WI, USA

This paper represents a vanguard analytical investigation on the role of adhesion molecules in regulating the size of enteric ganglia. This paper demonstrates that both *in vitro* and *in vivo*, Hu ^+ve^ enteric neural crest cells showing a neuronal phenotype tend to “sort out” within the centre of ganglia and adhere together while Hu ^-ve^ enteric neural crest cells show less adhesion and are located more in the periphery.

I have a few comments that I would like to be addressed in order to increase the clarity and impact of this manuscript for the reader.

1. In the Introduction the authors state “: Hypoganglionosis (fewer, smaller ganglia) is associated with persistent constipation…” Could they please provide a citation for this statement?
2. On page 2 the authors wrote. “Formation of each **the** NC-derived sympathetic ganglia, for example, relies on the scattered NC cells self-aggregating…” Please delete “the” from this statement.
3. On page 4 “The tubes were then incubated on a rotating platform (120 rpm, radius 1 cm) at 37 ^o^C. At t=0, 15, 30, 60 and 120 min.,” Please clarify the position that the tubes were incubated, vertical or horizontal?
4. On page 6. “HNK-1+ve sorted cells plated on fibronectin or laminin surfaces showed neural immunoreactivity, including SoxE (recognizes Sox9 and Sox10). In the description of the antibodies it states that this antibody also recognizes Sox8. Therefore, could Sox8 please be added within the parentheses.
5. In Fig. 1 . The y axis label reads cells per 100mm2, I think that this might be error and should be cells per 100 microns squared. Could the authors please amend this?
6. On Page 6. The authors wrote, “The sensitivity to EGTA showed involvement of Ca ^2+^-dependent (i.e. cadherin) mechanisms, but the residual aggregation indicated Ca ^2+^-independent mechanisms (such as NCAM) were operative as well, suggests that the initial phase of adhesion in these conditions was largely due to Ca ^2+^-independent adhesion.” This section could be made more clear with some revision. As the words residual aggregation suggest that it is a later time point and so maybe the removal of the word residual would make this sentence easier to read and understand what the authors are trying to say.
7. Figure 6 needs to be modified so that each of the fluorophores are shown separately, especially 6D. It would be useful for the reader to be informed where the optical section was taken from. Are we looking at a section from the surface or deeper within the aggregate as this changes how we can interpret what we are looking at.
8. Figure 6. It is not clear from visual inspection that the levels of NCAM are really different as stated “Labelling for NCAM showed that this adhesion molecule was most strongly expressed on the internal Hu ^+ve^ cells with lower immunoreactivity on the SoxE ^+ve^ cells,…”. The level is quite low between some Hu cells. Some quantification of the intensity of the NCAM staining would greatly improve the validity of the authors statements here.
9. Fig 7B. This image doesn’t support the assertion from the authors that the Hu cells are found in the center of the aggregates as many Hu ^+ve^ cells are found on outside in this image. Do the authors have an alternative image that could be used to demonstrate their conclusions? Alternatively could they make sure that they state the proportion of aggregates that had Hu ^+ve^ cells on their surface.
10. On page 10 the authors wrote, "This shows that the outer surface of pre-formed aggregates is relatively non-adhesive, and is consistent with the observation that the outer surface of the peripheral cells of aggregates showed low immunoreactivity for CAMs (Figures 6D, E).” The outer surface of the aggregates must be sticky at initial stages or the aggregates would not have increased in size during these stages since the authors have already shown us that aggregate formation at the early stages is not being regulated by cell proliferation.  Could the authors please amend this statement to ensure that it is a true reflection of what is occurring within their experiments?
11. On page 11. “Culturing these cells in Terasaki wells confirmed this was the case, both populations had SoxE+ve cells but HuC/D+ve cells with E-C8+ve, Tuj1+ve neurites occurred only in the NCAM high fraction.” It would be really good if the authors could show this data. If this is not possible then could they please state, data not shown here. 
12. Fig 9A. “Box plots show the first quartile to inter quartile range and whiskers show minimum and maximum range of data, while the median is represented by a vertical line.”  The authors are very imaginative but some readers don’t shave. Please change to vertical lines show minimum and maximum range of data respectively, while the median is represented by a horizontal line.
13. On page 11 the authors state, “Interestingly, the internal structure of the aggregates was strikingly different: SoxE+/Hu-ve cells and SoxE-/Hu+ve neurons were mixed not segregated (Figure 9B inset)”. Please add “in the DRG” at the end of this sentence.

We have read this submission. We believe that we have an appropriate level of expertise to confirm that it is of an acceptable scientific standard.
